# Genome-wide association analysis identifies loci governing mercury accumulation in maize

**DOI:** 10.1038/s41598-017-00189-6

**Published:** 2017-03-21

**Authors:** Zhan Zhao, Zhongjun Fu, Yanan Lin, Hao Chen, Kun liu, Xiaolong Xing, Zonghua Liu, Weihua Li, Jihua Tang

**Affiliations:** 1 Key Laboratory of Wheat and Maize Crops Science, Collaborative Innovation Center of Henan Grain Crops, College of Agronomy, Henan Agricultural University, Zhengzhou, 450002 China; 2 Maize Research Institute, Chongqing Academy of Agricultural Sciences, Chongqing, 401329 China; 3 Hubei Collaborative Innovation Center for Grain Industry, Yangtze University, Jingzhou, 434025 China

## Abstract

Owing to the rapid development of urbanisation and industrialisation, heavy metal pollution has become a widespread environmental problem. Maize planted on mercury (Hg)-polluted soil can absorb and accumulate Hg in its edible parts, posing a potential threat to human health. To understand the genetic mechanism of Hg accumulation in maize, we performed a genome-wide association study using a mixed linear model on an association population consisting of 230 maize inbred lines with abundant genetic variation. The order of relative Hg concentrations in different maize tissues was as follows: leaves > bracts > stems > axes > kernels. Combined two locations, a total of 37 significant single-nucleotide polymorphisms (SNPs) associated with kernels, 12 with axes, 13 with stems, 27 with bracts and 23 with leaves were detected with *p* < 0.0001. Each significant SNP was calculated and the SNPs significant associated with kernels, axes, stems, bracts and leaves explained 6.96%–10.56%, 7.19%–15.87%, 7.11%–10.19%, 7.16%–8.71% and 6.91%–9.17% of the phenotypic variation, respectively. Among the significant SNPs, nine co-localised with previously detected quantitative trait loci. This study will aid in the selection of Hg-accumulation inbred lines that satisfy the needs for pollution-safe cultivars and maintaining maize production.

## Introduction

Pollution-safe cultivar, which refers to the use of cultivars that accumulate a very low level of a specific pollutant, have been proposed to be a strategy to ensure the crop remains safe for human consumption, even when grown in contaminated soil^1^. Owing to rapid urbanisation and industrialisation, heavy metal pollution has become a widespread environmental problem^2^. Many processes, such as the application of agrochemicals (fertilisers, pesticides and animal manures), sewage irritation and industrial pollution (including from nonferrous metal, ceramics, printing and dyeing, electroplate and chemical adhesive industries), have emitted heavy metals into soils^3^. McLaughlin *et al*.^4^ reported that metals could be transferred into the roots of plants through the soil pore water in the form of dissolved ions. The heavy metals can then accumulate in the edible part of plants and easily enter the human body through the food chain, which results in an increased risk of disease^5–7^. Mercury (Hg) is one of the most toxic heavy metals^8, 9^. According to the Chinese National Standard for Soil Environmental Quality, the Hg concentration in soils is divided into three classes: in Class I, the Hg concentration is under 0.15 mg kg^−1^ in soils, which is the natural background value; in Class II, the Hg concentration is under 1 mg kg^−1^, which is the upper acceptable limit for agricultural soils; and in Class III, the Hg concentration is under 1.5 mg kg^−1^ in soils, which is the limit for the normal growth of plants^10^. According to Lin *et al*.^11^, the background Hg levels of soils in China range from 0.02 to 0.20 mg kg^−1^. However, the soil Hg content in many parts of China far exceeds its background value. For example, the soil Hg content at the site of the Wuchuan mine in China is as high as 24 mg kg^−1^ ^9^.

A high soil Hg content can impact plant growth and development when absorbed by plants. Seed injury to cereals caused by organomercury can inhibit cell division during seed germination, and the elongation of *Oryza sativa* seedlings is also inhibited by high Hg concentration^12^. Hg can cause a large and rapid reduction in the hydraulic conductivity of roots, which results in the inhibition of aquaporin functions^13, 14^, and higher Hg concentrations have a toxic effect on root growth in alfalfa by inducing oxidative stress^15^. Additionally, high Hg doses can affect the absorption and evaporation of water, and decrease the chlorophyll content and photosynthetic efficiency^16–18^. Importantly, the soil Hg absorbed by crops can also be transported into the human body through the food chain^19^. Hg, in the form of methylethylmercury, absorbed by the human body can accumulate in the brain, eventually causing neurotoxic effects and nerve-related diseases, such as autism, attention deficit disorder and mental retardation, and death^20, 21^.

Many studies have investigated the physiological and biochemical responses to Hg intoxication. Hg also inhibits the 5-amino levulinic acid dehydratase activity in the cells of maize leaves, thereby affecting the synthesis of chlorophyll^22^, and inhibiting plant growth and development^23^. Yu *et al*. first identified three quantitative trait loci (QTLs) for Hg accumulation and tolerance at the seedling stage in rice using a doubled haploid population^24^. Wang *et al*. also detected three QTLs for Hg tolerance at the seedling stage in rice using a recombinant inbred population^25^. Fu *et al*. found 23 QTLs for Hg accumulation in five maize tissues using a recombinant inbred population^26^. In addition to these QTLs, genes closely related to Hg accumulation and tolerance have also been reported. In *Arabidopsis thaliana*, *merApe9* transgenic plants are more tolerant to Hg at the seedling stage and during the flowering period^27^. The overexpression of *HO-1* in algae also resulted in a high tolerance to Hg exposure and a reduced Hg accumulation^28^. The overexpression of *BnHO-1* in *Brassica napus* resulted in a reduced Hg accumulation in the transgenic plants^29^. *MTH1745* transgenic rice has an enhanced Hg tolerance^30^.

Genome-wide association studies (GWASs) are useful for identifying candidate loci associated with traits in animal and plant species^31, 32^. For example, the examination of maize oil biosynthesis identified 74 loci significantly associated with kernel oil concentration and fatty acid composition in a GWAS using 1 million single-nucleotide polymorphisms (SNPs) characterised in 368 maize inbred lines^33^. Furthermore, GWAS and QTL mapping have been found to be complementary and to overcome each other's limitations in *Arabidopsis* ^34, 35^. In soya bean, the genotyping by sequencing-GWAS approach has been used to identify loci governing eight agronomic traits and was validated by QTL mapping^36^.

Maize is an important grain and feed crop for humans and animals. Hg accumulation in plants through the plant–soil system can cause toxicity that affects both plant growth and human health through the food chain^12^. Thus, maize planted on Hg-polluted soil can absorb and accumulate Hg in its edible parts, posing a potential threat to human health. Although some QTLs and genes regulating the accumulation of, and tolerance to, Hg have been reported previously^24–30^, knowledge of the genetic basis for Hg accumulation in maize remains limited. In the present study, an association population consisting of 230 maize inbred lines was evaluated at two locations having different soil Hg concentrations. The main purpose of this study was to detect SNPs that are significantly associated with Hg accumulation in five maize tissues, which may aid in the selection of elite inbred lines with low Hg-accumulation capabilities in the kernels and high accumulation capabilities in the leaves, to maintain maize production.

## Results

### Performance of the measured traits

**Figure 1:**
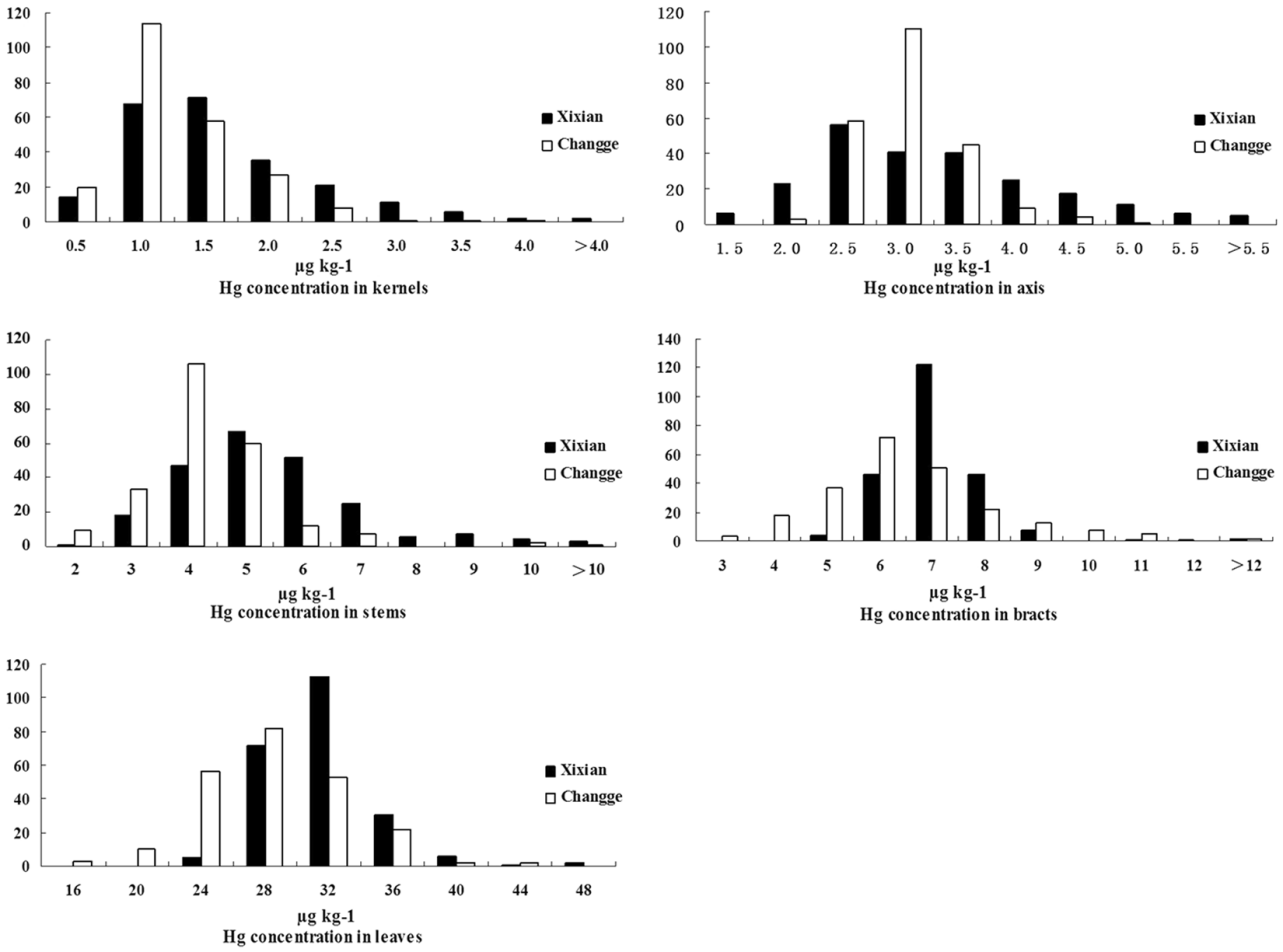
Histogram of Hg concentrations in five maize tissues in the association population.

In the association population, the Hg content in all five of the maize tissues tested was much higher at the Xixian location compared with the corresponding tissues at Changge (Table 1, Fig. 1). At Xixian, the average Hg contents in the kernels, axes, stems, bracts and leaves were 1.39, 3.04, 4.94, 6.62 and 29.46 µg kg^−1^, respectively. At Changge, the average Hg contents in the kernels, axes, stems, bracts and leaves were 1.05, 2.79, 3.84, 6.01 and 26.49 µg kg^−1^, respectively. The Hg concentrations in the different maize tissues showed the same trend, kernels < axes < stems < bracts < leaves, at the two locations.

**Table 1:** Means and standard deviation (SD) values, variance components and heritability values of maize kernels, axes, stems, bracts and leaves.

Location	Tissue	Average ± SD	Range	Median	${{\boldsymbol{\sigma }}}_{{\bf{g}}}^{{\bf{2}}}$	${{\boldsymbol{\sigma }}}_{{\bf{ge}}}^{{\bf{2}}}$	*H* ^2c^(%)
		(µg kg^−1^)	(µg kg^−1^)	(µg kg^−1^)			
Xixian	Kernels	1.39 ± 0.79	0.15–6.50	1.23	0.71**		86.50
	Axes	3.04 ± 1.00	1.01–6.4	2.92	0.87**		80.28
	Stems	4.94 ± 1.72	1.93–14.39	4.64	3.19**		79.85
	Bracts	6.62 ± 1.01	4.61–12.78	6.49	3.17**		85.94
	Leaves	29.46 ± 3.37	20.50–46.30	29.26	24.85**		82.93
Changge	Kernels	1.05 ± 0.52	0.14–3.55	0.93	0.81**		88.55
	Axes	2.79 ± 0.46	1.82–4.86	2.76	2.98**		92.50
	Stems	3.84 ± 1.19	1.31–10.35	3.68	8.88**		95.38
	Bracts	6.01 ± 1.71	2.37–13.40	5.77	8.73**		97.39
	Leaves	26.49 ± 4.62	12.26–42.43	26.14	64.05**		98.67
Combined	Kernels					0.32**	77.79
	Axes					0.47**	87.20
	Stems					1.69**	85.35
	Bracts					1.36**	88.20
	Leaves					12.11**	85.97

^*^Significant at *p* < 0.05. **Significant at *p* < 0.01.

The Hg content in each maize tissue varied widely in the association population at the two locations. The Hg concentration in kernels varied from 0.15 to 6.50 µg kg^−1^ at Xixian and from 0.14 to 3.55 µg kg^−1^ at Changge. The Hg concentrations in axes, stems, bracts and leaves varied from 1.01 to 6.4 µg kg^−1^, 1.93 to 14.39 µg kg^−1^, 4.61 to 12.78 µg kg^−1^ and 20.50 to 46.30 µg kg^−1^, respectively, at Xixian; and from 1.82 to 4.86 µg kg^−1^, 1.31 to 10.35 µg kg^−1^, 2.37 to 13.40 µg kg^−1^ and 12.26 to 42.43 µg kg^−1^, respectively, at Changge.

An analysis of variance indicated that the Hg concentrations in the five measured tissues in the association population were significantly affected by environments, genotypes and genotype × environment interactions (Table 1), indicating that the soil Hg concentration is an important factor affecting the Hg contents in maize tissues. The heritability values of the Hg contents in kernels, axes, stems, bracts and leaves at Xixian were 86.50%, 80.82%, 79.85%, 85.94% and 82.93%, respectively; and 88.55%, 92.50%, 95.38%, 97.39% and 98.67%, respectively, at Changge. The high heritability in each tissue at both locations indicated that much of the phenotypic variance in the population was genetically controlled. Additionally, the correlations of the Hg contents in the five tissues at each location and the means of the Hg concentrations of the corresponding tissues at both locations were calculated using the Pearson correlation coefficient. At Xixian, the Hg concentrations between bracts and stems showed a significant relationship (Table 2), while at Changge, the kernel's Hg concentration had significant relationships with those of both bracts and axes. In the two locations combined, the mean of the bract's Hg content had significant relationships with those of both kernels and stem.

**Table 2:** Correlation coefficients among different tissues in the maize association population.

Location	Trait	Kernels	Axis	Stems	Bracts
Xixian	Kernels	1.00			
	Axes	−0.03	1.00		
	Stems	0.02	−0.04	1.00	
	Bracts	0.08	0.04	−0.13*	1.00
	Leaves	−0.13	−0.03	0.07	0.05
Changge	Kernels	1.00			
	Axes	−0.14*	1.00		
	Stems	−0.04	−0.09	1.00	
	Bracts	0.18**	0.01	−0.12	1.00
	Leaves	−0.02	−0.03	−0.03	−0.02
Combined	Kernels	1.00			
	Axes	−0.10	1.00		
	Stems	−0.01	−0.08	1.00	
	Bracts	0.15*	0.02	0.13*	1.00
	Leaves	−0.07	0.03	0.02	−0.004

^*^Significant at *p* < 0.05. **Significant at *p* < 0.01.

### Linkage disequilibrium (LD) in the association panel

**Figure 2:**
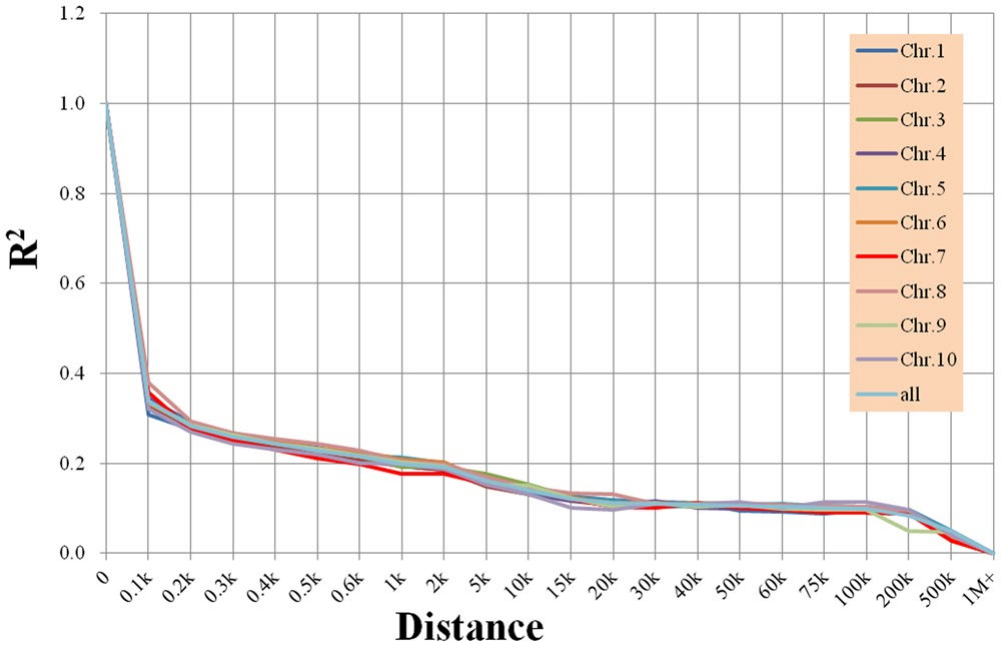
Linkage disequilibrium decay of each chromosome.

The genome-wide LD was calculated using the 522,744 SNPs (minor allelic frequency > 0.05), which were used as the input data (Fig. 2). LD decay varied from 50 kb to100 kb across different chromosomes at r = 0.1. The LD reached within 40–50 kb on chromosome 1, 50–60 kb on chromosome 2, 75–100 kb on chromosome 3, and 50–100 kb on the remaining chromosomes. The average LD decay across the entire genome was 50–100 kb (r = 0.1).

### GWAS

**Figure 3:**
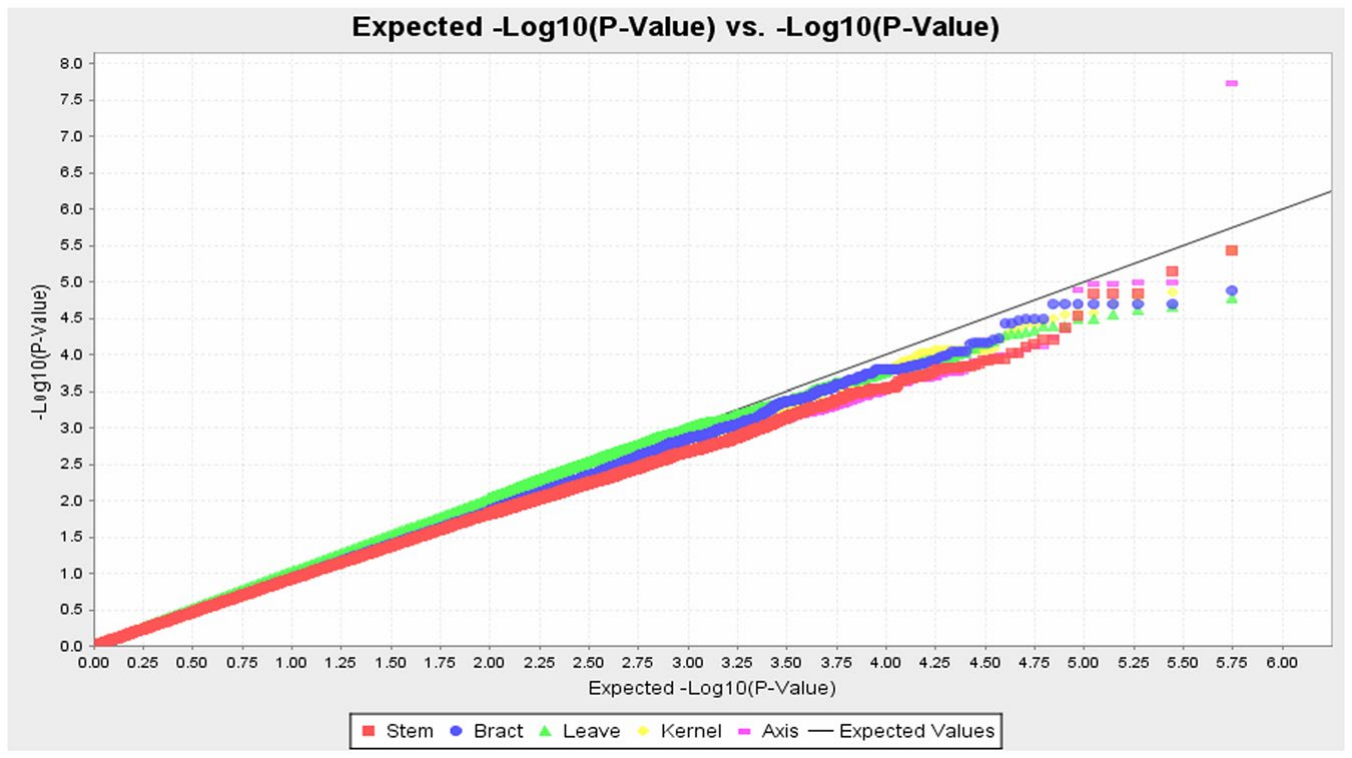
Quantile–quantile plot of the Hg contents in five maize tissues from two locations combined, as determined by a genome-wide association analysis.

**Figure 4:**
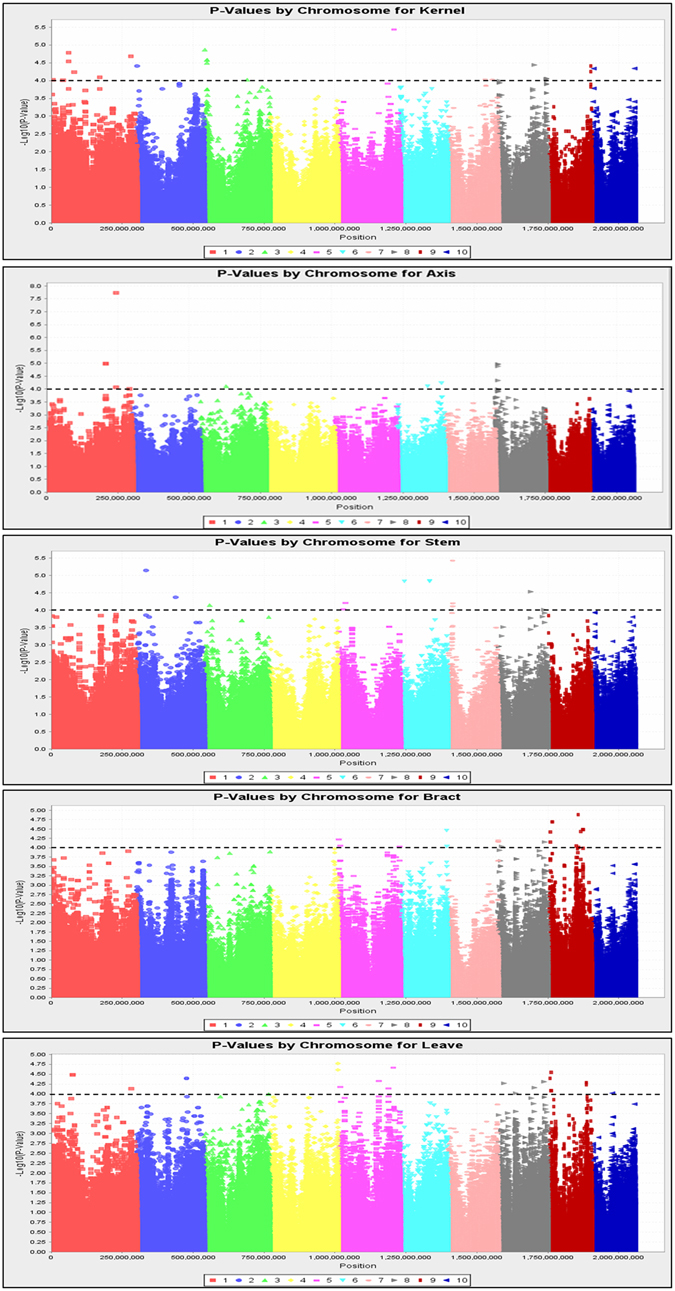
Manhattan plot of the Hg contents in five maize tissues from two locations combined, as determined by a genome-wide association analysis.

A total of 230 inbred lines were characterised phenotypically across different tissues at two different sites. Principal components (PCs) were used to control the population structure. A principal component analysis (PCA) showed that the inbred lines used in this study could be separated well by PC1 and PC2 (Fig. S1). A GWAS was performed for the Hg contents in five maize tissues using a mixed linear model (Figs 3 and 4). A total of 37 significant SNPs that associated with kernels, 12 with axes, 13 with stems, 27 with bracts and 23 with leaves were detected with *p* < 0.0001, which explained 6.96%–10.56%, 7.19%–15.87%, 7.11%–10.19%, 7.16%–8.71% and 6.91%–9.17% of the phenotypic variation for kernels, axes, stems, bracts and leaves, respectively (Table 3). All of these significant SNPs were distributed over 10 chromosomes. In the association population, the most significant SNPs for kernels, axes, stems, bracts and leaves were chr5. S_196250608, chr1.S_242708944, chr7. S_15426996, chr9. S_105527159 and chr4. S_239906146, respectively, located on chromosome 5, 1, 7, 9 and 4, respectively, explaining 10.56%, 15.86%, 10.19%, 8.71% and 9.17% of the phenotypic variation, respectively. The quantile–quantile plots were determined and indicated that population structure was well controlled by PCA and Kinship of each tissue.

**Table 3:** A total of 111 significantly associated SNPs in maize detected by GWAS.

Trait	Marker	Chr	Position	p	MarkerR2
Axis	chr1.S_206512615	1	206512615	1.02E-05	0.09515
Axis	chr1.S_206513913	1	206513913	1.02E-05	0.09515
Axis	chr1.S_242708944	1	242708944	1.86E-08	0.15866
Axis	chr1.S_242710182	1	242710182	8.44E-05	0.07368
Axis	chr1.S_289788237	1	289788237	9.97E-05	0.07452
Axis	chr3.S_91357195	3	91357195	7.87E-05	0.0719
Axis	chr6.S_107179621	6	107179621	7.66E-05	0.07735
Axis	chr6.S_155668107	6	155668107	5.75E-05	0.08035
Axis	chr8.S_8290083	8	8290083	1.30E-05	0.09392
Axis	chr8.S_8290137	8	8290137	4.65E-05	0.0831
Axis	chr8.S_8290190	8	8290190	1.09E-05	0.09618
Axis	chr8.S_8290329	8	8290329	1.09E-05	0.09618
Bracts	chr5.S_921802	5	921802	6.08E-05	0.08303
Bracts	chr5.S_6141833	5	6141833	8.92E-05	0.07592
Bracts	chr5.S_215965598	5	215965598	9.25E-05	0.08033
Bracts	chr6.S_163920776	6	163920776	3.44E-05	0.0852
Bracts	chr6.S_164498297	6	164498297	9.20E-05	0.08036
Bracts	chr7.S_176391688	7	176391688	6.31E-05	0.08489
Bracts	chr7.S_176391721	7	176391721	6.86E-05	0.08261
Bracts	chr7.S_176391728	7	176391728	6.86E-05	0.08261
Bracts	chr7.S_176391743	7	176391743	6.86E-05	0.08261
Bracts	chr8.S_13211217	8	13211217	9.29E-05	0.07336
Bracts	chr8.S_163741736	8	163741736	6.97E-05	0.07291
Bracts	chr9.S_7959782	9	7959782	7.05E-05	0.07163
Bracts	SYN39067	9	7960037	3.78E-05	0.08324
Bracts	chr9.S_14548691	9	14548691	2.04E-05	0.08317
Bracts	chr9.S_14548741	9	14548741	2.04E-05	0.08317
Bracts	chr9.S_14548749	9	14548749	2.04E-05	0.08317
Bracts	chr9.S_14548854	9	14548854	2.04E-05	0.08317
Bracts	chr9.S_14548892	9	14548892	2.04E-05	0.08317
Bracts	chr9.S_14548893	9	14548893	2.04E-05	0.08317
Bracts	chr9.S_14549008	9	14549008	2.04E-05	0.08317
Bracts	PZE-109058129	9	99366524	8.93E-05	0.07311
Bracts	chr9.S_105527159	9	105527159	1.31E-05	0.08712
Bracts	chr9.S_107622070	9	107622070	9.82E-05	0.07655
Bracts	chr9.S_115321300	9	115321300	3.74E-05	0.08445
Bracts	chr9.S_123408721	9	123408721	3.29E-05	0.08267
Bracts	chr9.S_123408722	9	123408722	3.29E-05	0.08267
Bracts	chr9.S_123408723	9	123408723	3.29E-05	0.08267
Kernels	chr1.S_6170206	1	6170206	9.58E-05	0.07241
Kernels	SYN9128	1	40355622	9.72E-05	0.06959
Kernels	chr1.S_60754581	1	60754581	2.85E-05	0.08256
Kernels	chr1.S_60754606	1	60754606	1.65E-05	0.08883
Kernels	chr1.S_80189675	1	80189675	5.84E-05	0.07783
Kernels	chr1.S_170305135	1	170305135	8.16E-05	0.0764
Kernels	PZE-101231477	1	280342748	2.06E-05	0.08907
Kernels	chr2.S_511207	2	511207	3.91E-05	0.08308
Kernels	chr3.S_2940421	3	2940421	1.38E-05	0.08861
Kernels	chr3.S_9935521	3	9935521	3.19E-05	0.08327
Kernels	chr3.S_9936025	3	9936025	2.63E-05	0.08496
Kernels	chr3.S_9936028	3	9936028	2.63E-05	0.08496
Kernels	chr3.S_151968221	3	151968221	9.42E-05	0.07339
Kernels	chr5.S_196250608	5	196250608	3.64E-06	0.10561
Kernels	chr7.S_130427527	7	130427527	9.31E-05	0.0753
Kernels	chr7.S_155614940	7	155614940	9.39E-05	0.07115
Kernels	chr8.S_128481917	8	128481917	3.64E-05	0.08179
Kernels	chr8.S_170833808	8	170833808	8.82E-05	0.07589
Kernels	chr8.S_170846071	8	170846071	8.82E-05	0.07589
Kernels	chr8.S_170846085	8	170846085	8.82E-05	0.07589
Kernels	chr8.S_170846136	8	170846136	8.82E-05	0.07589
Kernels	chr8.S_170847326	8	170847326	8.82E-05	0.07589
Kernels	chr8.S_170847485	8	170847485	8.82E-05	0.07589
Kernels	chr8.S_170847489	8	170847489	8.82E-05	0.07589
Kernels	chr8.S_170850217	8	170850217	8.82E-05	0.07589
Kernels	chr8.S_170850473	8	170850473	8.82E-05	0.07589
Kernels	chr8.S_170851375	8	170851375	8.82E-05	0.07589
Kernels	chr8.S_170851664	8	170851664	8.82E-05	0.07589
Kernels	chr8.S_170852211	8	170852211	8.82E-05	0.07589
Kernels	chr8.S_170852659	8	170852659	8.82E-05	0.07589
Kernels	chr8.S_170853700	8	170853700	8.82E-05	0.07589
Kernels	chr8.S_170853730	8	170853730	8.82E-05	0.07589
Kernels	chr8.S_170854083	8	170854083	8.82E-05	0.07589
Kernels	chr9.S_150656046	9	150656046	5.62E-05	0.08374
Kernels	chr9.S_150707606	9	150707606	3.89E-05	0.08631
Kernels	chr10.S_4682197	10	4682197	4.63E-05	0.08518
Kernels	chr10.S_148974418	10	148974418	4.52E-05	0.09246
Leaves	chr1.S_74416852	1	74416852	3.26E-05	0.07957
Leaves	chr1.S_74416853	1	74416853	3.26E-05	0.07957
Leaves	chr1.S_281320598	1	281320598	7.28E-05	0.07679
Leaves	chr2.S_175260655	2	175260655	4.08E-05	0.08417
Leaves	chr2.S_175260694	2	175260694	4.08E-05	0.08417
Leaves	chr4.S_239906146	4	239906146	1.69E-05	0.09172
Leaves	chr4.S_239929354	4	239929354	2.48E-05	0.08594
Leaves	chr5.S_6442336	5	6442336	6.67E-05	0.08171
Leaves	chr5.S_143198459	5	143198459	4.67E-05	0.07701
Leaves	chr5.S_176898742	5	176898742	7.22E-05	0.0749
Leaves	chr5.S_193558440	5	193558440	2.17E-05	0.08504
Leaves	chr8.S_20488572	8	20488572	5.42E-05	0.07478
Leaves	PZE-108038334	8	62522497	9.68E-05	0.07302
Leaves	chr8.S_128751246	8	128751246	6.89E-05	0.07321
Leaves	chr8.S_162579911	8	162579911	4.87E-05	0.07971
Leaves	chr9.S_7577801	9	7577801	4.06E-05	0.07991
Leaves	chr9.S_11233162	9	11233162	2.83E-05	0.08324
Leaves	chr9.S_11234080	9	11234080	8.19E-05	0.07324
Leaves	chr9.S_11234108	9	11234108	8.19E-05	0.07324
Leaves	chr9.S_134237753	9	134237753	5.17E-05	0.07637
Leaves	chr9.S_134237754	9	134237754	5.17E-05	0.07637
Leaves	chr9.S_134293724	9	134293724	5.89E-05	0.08125
Leaves	chr10.S_70916728	10	70916728	9.56E-05	0.06905
Stems	chr2.S_32492547	2	32492547	7.12E-06	0.09288
Stems	PZE-102107302	2	136419732	4.28E-05	0.08288
Stems	chr3.S_19784204	3	19784204	7.25E-05	0.07661
Stems	chr5.S_16425840	5	16425840	9.41E-05	0.07353
Stems	chr5.S_24609422	5	24609422	6.21E-05	0.07529
Stems	chr6.S_15236064	6	15236064	1.45E-05	0.08923
Stems	chr6.S_103987005	6	103987005	1.48E-05	0.09155
Stems	chr6.S_103987029	6	103987029	1.42E-05	0.09227
Stems	chr7.S_15426996	7	15426996	3.72E-06	0.10193
Stems	chr7.S_16096718	7	16096718	7.75E-05	0.07334
Stems	chr7.S_16096719	7	16096719	6.31E-05	0.07521
Stems	chr8.S_115638001	8	115638001	2.90E-05	0.08065
Stems	chr8.S_161469759	8	161469759	9.73E-05	0.07114

The overall LD decay for the entire genome in this panel was 100 kb. Given the extent of average LD, we feel confident that a 200 kb window centered on each significant SNP has a good chance to capture the gene of interest.

## Discussion

Hg poisoning, as the result of environmental pollution, has become a problem. In rice, the Hg content in different tissues follows the trend: root > stalk > leaf > husk > seed^37^. In maize, Liu *et al*.^38^ reported that the Hg levels in different tissues were different, having the following trend: root > leaf > stalk > grain. Fu *et al*.^26^ found a similar distribution of the Hg content, leaves > bracts > stems > axes > kernels, in maize. The same trend for Hg concentrations in different maize tissues was also found in the present study (Table 1). The similar distribution of the Hg content across different maize tissues indicated that a common regulatory mechanism might exist in the Hg-accumulation process.

In crop breeding, QTL mapping for important traits is a most common approach, providing the basis for marker-assisted selection. However, QTL detection is a traditional genotyping method and is laborious and time-consuming, and QTLs are low-density markers that map with low resolutions because of the limited recombination numbers. With the advent of next-generation sequencing technologies, GWASs have become a powerful genetics-based strategy to explore allelic variation with a broader scope. GWASs can save time and labour, increase the detectable range of natural variation and improve the resolution of QTL mapping^39, 40^. In plants, GWASs have been used to identify many loci for complex traits, including drought tolerance^41^, and seed oil^36^ and protein contents^41^. In the present study, 11 significant SNPs associated with Hg accumulation co-localised with six previously reported QTL intervals^26^ (Table 4). On chromosome 7, one significant SNP (chr7.S_130427527) associated with the Hg content in kernels was detected within the *qAHC7* QTL interval located in bin7.03. A total of five significant SNPs on chromosome 8, which associated with the Hg contents in kernels (chr8.S_128481917), stems (chr8.S_161469759), bracts (chr8.S_163741736) and leaves (chr8.S_128751246 and chr8.S_162579911), co-localised with the previously reported QTL named *qBHC8a*. The other significant SNP (PZE-108038334) on chromosome 8 co-localised with the *qSHC8b* QTL located in bin8.03. On chromosome 9, three significantly associated SNPs observed within the region between 105.527 and 115.321 Mb co-localised with the reported QTL *qKHC9b/qBHC9*. The other significant SNP (PZE-109058129) on chromosome 9 co-localised with the *qKHC9a* QTL located in bin9.03.

**Table 4:** Identification of maize loci using a GWAS and previous QTLs.

Location	Tissue	Loci	Chr	Position	P	DF	MarkerR^2^	bin	QTL
Combined	Kernels	chr7.S_130427527	7	130427527	9.31E-05	213	0.075	7.03	*qAHC7*
Combined	Kernels	chr8.S_128481917	8	128481917	3.64E-05	221	0.082	8.05	*qBHC8a*
Combined	Stems	chr8.S_161469759	8	161469759	9.73E-05	215	0.071	8.06	*qBHC8a*
Combined	Bracts	PZE-109058129	9	99366524	8.93E-05	211	0.073	9.03	*qKHC9a*
Combined	Bracts	chr9.S_105527159	9	105527159	1.31E-05	227	0.087	9.04	*qKHC9b/qBHC9*
Combined	Bracts	chr9.S_107622070	9	107622070	9.82E-05	214	0.077	9.04	*qKHC9b/qBHC9*
Combined	Bracts	chr9.S_115321300	9	115321300	3.74E-05	222	0.084	9.04	*qKHC9b/qBHC9*
Combined	Bracts	chr9.S_123408721	9	123408721	3.29E-05	218	0.083	9.04	*qBHC8a*
Combined	Leaves	PZE-108038334	8	62522497	9.68E-05	221	0.073	8.03	*qSHC8b*
Combined	Leaves	chr8.S_128751246	8	128751246	6.89E-05	223	0.073	8.05	*qBHC8a*
Combined	Leaves	chr8.S_162579911	8	162579911	4.87E-05	210	0.080	8.06	*qBHC8a*

In the present study, more loci were detected through the GWAS than through linkage mapping, possibly because of the reduced QTL detection efficiency and the smaller number of molecular markers used in linkage mapping. A combination of linkage mapping and GWAS would promote the analysis of complex quantitative traits. The loci identified by the integration of GWAS and QTL mapping would provide useful reference information for studies on the functional verification of Hg accumulation.

Because maize is a food, feed and industrial crop, increasing its grain yield is an important breeding goal^42^, and is also important to ensure the quality of maize grain as worldwide demand for food increases. Heavy metal pollution in soils has become a major issue globally^43^, and the heavy metals absorbed by crops could affect grain quality. Zhang *et al*.^44^ reported that it was necessary and feasible to select and plant low heavy metal accumulation varieties in contaminated soil, which could reduce the heavy metal content of the edible crop parts. The strategy of breeding pollution-safe cultivars, in which heavy metals in contaminated soil could accumulate at sufficiently low levels in the edible parts of crops for safe consumption^1^, has been applied. Hg usually accumulates to a significantly higher level in leaves than in kernels^25, 39, 40^, and this was corroborated in the present study. For example, the mean Hg concentrations in leaves were 29.46 µg kg^−1^ at Xixian and 26.49 µg kg^−1^ at Changge, which far exceeds that in kernels (1.39 µg kg^−1^ at Xixian and 1.05 µg kg^−1^ at Changge). In addition, the Pearson correlation coefficient indicates that there is no significant relationship between the Hg contents in leaves and kernels. The results imply that inbred lines with high accumulations of Hg in leaves and low accumulations of Hg in kernels could be used as parents for selecting elite hybrids, which would be helpful both for soil remediation and for the assurance of food safety.

## Materials and Methods

### Plant materials and SNP markers

A total of 298 maize inbred lines, which came from temperate, tropical and subtropical zones, were used in this study. After removing materials with higher heterozygosity and loss rates, 230 inbred lines were selected to constitute the association population, among which 151 inbred lines came from the temperate zone, and 79 came from tropical and subtropical zones (Supplementary Table S1)^45^. The selected inbred lines were genotyped in this study using two genotyping platforms (RNA sequencing and a SNP array) containing 556,809 SNPs according to the method described by Yang *et al*.^45^ The SNP data is available from http://www.maizego.org/Resources.html.

### Plant treatments and soil conditions

The field trials were conducted in 2012 in Xixian (E 114°72′, N 32°35′) and Changge (E 113°34′, N 34°09′) Counties, which are located in northern China, with average temperatures of 15.2 and 14.3 °C, respectively, and rainfalls of 873.8 and 462.8 mm, respectively. The maize association population was grown in a randomised complete block design with three replicates at each location. Each plot included 15 plants planted 0.67 m apart in a single row 4 m long, allowing a final plant density of 67,500 plants per hectare. At the Xixian location, because of irrigation with Hg-rich surface water, the soil Hg concentration (457.57 ± 31.30 µg kg^−1^, pH 6.5) was much higher than at Changge (345.40 ± 22.24 µg kg^−1^, pH 6.5). The soil Hg concentrations at the two locations were higher than in Class I, according to the Chinese national standards related to Hg soil concentration rankings^10^.

### Determining the Hg concentrations in maize tissues

Five consecutive plants from each plot were harvested for further analyses when they reached physiological maturity. After the collected plant materials were dried, they were dissected into five parts: kernels, axes, stems, bracts and leaves. Each part of the plant was ground into a fine powder using a mortar and pestle. Powdered samples (0.5 g) were digested with 5 mL HNO_3_/HClO_4_ (80/20 v/v) in polypropylene tubes using a heating block (AIM500 Digestion System, A.I. Scientific, Australia). Then, the Hg concentrations in the different plant materials were determined using atomic fluorescence spectrometry (AFS-3000, Beijing Haiguang Analytical Instrument Co., Beijing, China) (Supplementary Table S2). Data were analysed using a two-way analysis of variance with the IBM SPSS Statistics package, and broad-sense heritability was calculated according to the method developed by Knapp *et al*.^46^.

### GWAS

SNPs with more than 12% missing data and a minor allele frequency < 5% were excluded, leaving 522,744 SNPs for further analyses. The LD between SNPs on each chromosome was estimated with r^2^ using TASSEL 5.0^47^. The PCs and the kinship matrix were also determined using TASSEL 5.0. A mixed linear model with the obtained SNPs, PCs, kinship and the means of the Hg contents was established for the GWAS. The relative distribution of −log_10_ *p*-values was observed for each SNP association and compared individually with the expected distribution using a quantile–quantile and manhattan plot. The adjusted *p*-value threshold of significance for each trait was corrected. SNP loci in significant LD regions were identified by revealing the significant contributions to phenotypic variation of the agronomic traits with the highest magnitude of marker trait-association and lowest adjusted *p*-values (threshold *p* < 1 × 10^−4^).

### Analysis of candidate genes

The reported genome sequence of maize B73 provides a useful reference database for candidate gene analyses. According to Lawrence *et al*.^48^, probes of ~120 bp containing the SNPs associated with Hg accumulation in different maize tissues were used for comparisons with the maize B73 genome. Based on the LD decay, a 200-kb window for the significant SNPs (100 kb upstream and downstream of the lead SNP) was selected to identify the candidate genes. Genes within the region were identified based on the positions of the closest flanking significant SNPs (*p* < 1 × 10^−4^). The candidate genes were obtained using the BLASTN algorithm (http://blast.ncbi.nlm.nih.gov/) and annotated against the Gene Ontology database (http://www.geneontology.org/) for functional annotations.

## Electronic supplementary material

Supplementary information file
