# Supplementary material for: Genome-wide association analysis identifies loci governing mercury accumulation in maize
Source: Sci Rep. 2017 Mar 21;7:247. doi: 10.1038/s41598-017-00189-6 (PMC5427852; doi:10.1038/s41598-017-00189-6)
Supplement: Supplementary file 1 — Supplementary information file [file 41598_2017_189_MOESM1_ESM.pdf]

# Supplementary information file

## Genome-wide association analysis identifies loci governing Hg accumulation in maize

Zhan Zhao[1※], Zhongjun Fu[1,2※], Yanan Lin[1], Hao Chen[1], Kun liu[1], Xiaolong Xing[1], Zonghua Liu[1],Weihua Li[1*], Jihua Tang[1,3*]

[1]Key Laboratory of Wheat and Maize Crops Science, Collaborative Innovation Center of Henan Grain Crops, College of Agronomy, Henan Agricultural University, Zhengzhou 450002, China

[2]Maize Research Institute, Chongqing Academy of Agricultural Sciences, Chongqing 401329, China

[3] Hubei Collaborative Innovation Center for Grain Industry, Yangtze University, Jingzhou, 434025, China

[※]The authors contributed equally to this work

[*]Corresponding author

Supplemental Figure Legends

Figure S1. Principal components analysis (PCA) for genome-wide association study population.

Figure S1

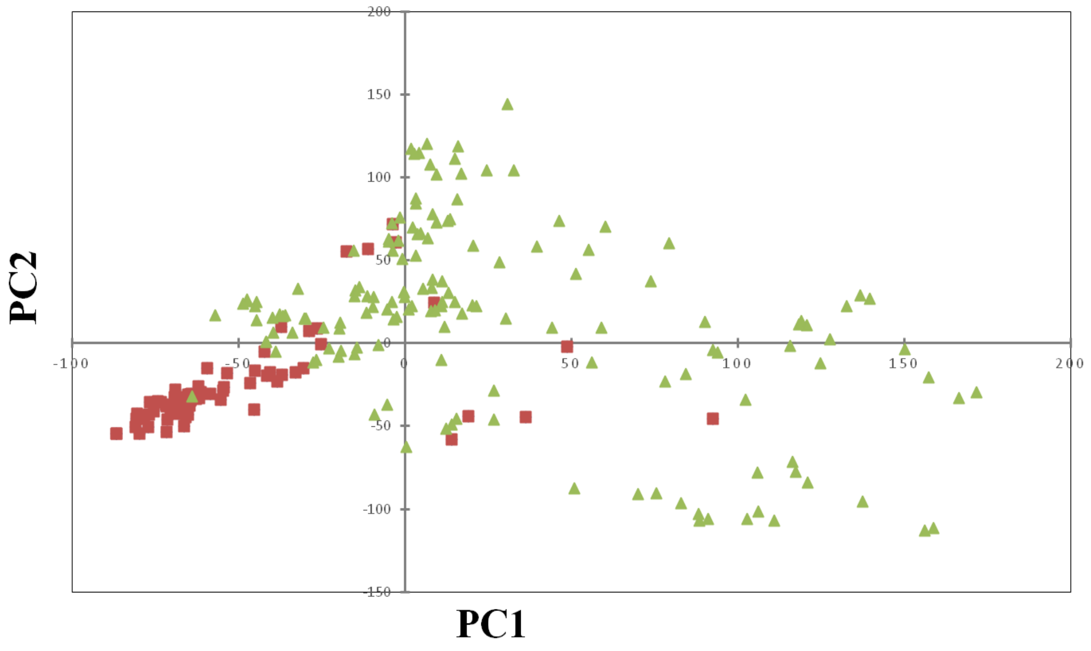

Table S1. Materials of the association population.

| number | The materials | adaptation |
|---|---|---|
| 1 | CML115 | tropical/subtropical |
| 2 | CML116 | tropical/subtropical |
| 3 | CML118 | tropical/subtropical |
| 4 | CML121 | tropical/subtropical |
| 5 | CML122 | tropical/subtropical |
| 6 | CML130 | tropical/subtropical |
| 7 | CML139 | tropical/subtropical |
| 8 | CML163 | tropical/subtropical |
| 9 | CML165 | tropical/subtropical |
| 10 | CML170 | tropical/subtropical |
| 11 | CML290 | tropical/subtropical |
| 12 | CML31 | tropical/subtropical |
| 13 | CML325 | tropical/subtropical |
| 14 | CML338 | tropical/subtropical |
| 15 | CML423 | tropical/subtropical |
| 16 | CML304 | tropical/subtropical |
| 17 | CML479 | tropical/subtropical |
| 18 | CIMBL1 | tropical/subtropical |
| 19 | CIMBL2 | tropical/subtropical |
| 20 | CIMBL8 | tropical/subtropical |
| 21 | CIMBL11 | tropical/subtropical |
| 22 | CIMBL12 | tropical/subtropical |
| 23 | CIMBL21 | tropical/subtropical |
| 24 | CIMBL23 | tropical/subtropical |
| 25 | CIMBL30 | tropical/subtropical |
| 26 | CIMBL32 | tropical/subtropical |
| 27 | CIMBL34 | tropical/subtropical |
| 28 | CIMBL38 | tropical/subtropical |
| 29 | CIMBL42 | tropical/subtropical |
| 30 | CIMBL45 | tropical/subtropical |
| 31 | CIMBL48 | tropical/subtropical |
| 32 | CIMBL49 | tropical/subtropical |
| 33 | CIMBL57 | tropical/subtropical |
| 34 | CIMBL58 | tropical/subtropical |
| 35 | CIMBL59 | tropical/subtropical |
| 36 | CIMBL60 | tropical/subtropical |
| 37 | CIMBL67 | tropical/subtropical |
| 38 | CIMBL75 | tropical/subtropical |
| 39 | CIMBL77 | tropical/subtropical |
| 40 | CIMBL80 | tropical/subtropical |
| 41 | CIMBL84 | tropical/subtropical |
| 42 | CIMBL85 | tropical/subtropical |

| | | |
|---|---|---|
| 43 | CIMBL86 | tropical/subtropical |
| 44 | CIMBL87 | tropical/subtropical |
| 45 | CIMBL88 | tropical/subtropical |
| 46 | CIMBL91 | tropical/subtropical |
| 47 | CIMBL92 | tropical/subtropical |
| 48 | CIMBL93 | tropical/subtropical |
| 49 | CIMBL96 | tropical/subtropical |
| 50 | CIMBL105 | tropical/subtropical |
| 51 | CIMBL106 | tropical/subtropical |
| 52 | CIMBL109 | tropical/subtropical |
| 53 | CIMBL110 | tropical/subtropical |
| 54 | CIMBL112 | tropical/subtropical |
| 55 | CIMBL117 | tropical/subtropical |
| 56 | CIMBL120 | tropical/subtropical |
| 57 | CIMBL123 | tropical/subtropical |
| 58 | CIMBL124 | tropical/subtropical |
| 59 | CIMBL127 | tropical/subtropical |
| 60 | CIMBL133 | tropical/subtropical |
| 61 | CIMBL134 | tropical/subtropical |
| 62 | CIMBL136 | tropical/subtropical |
| 63 | CIMBL138 | tropical/subtropical |
| 64 | CIMBL140 | tropical/subtropical |
| 65 | CIMBL142 | tropical/subtropical |
| 66 | CIMBL143 | tropical/subtropical |
| 67 | CIMBL144 | tropical/subtropical |
| 68 | CIMBL146 | tropical/subtropical |
| 69 | CIMBL150 | tropical/subtropical |
| 70 | CIMBL151 | tropical/subtropical |
| 71 | CIMBL153 | tropical/subtropical |
| 72 | CIMBL154 | tropical/subtropical |
| 73 | CIMBL157 | tropical/subtropical |
| 74 | By804 | temperate |
| 75 | By807 | temperate |
| 76 | By809 | temperate |
| 77 | By813 | temperate |
| 78 | By815 | temperate |
| 79 | By843 | temperate |
| 80 | By855 | temperate |
| 81 | By4839 | temperate |
| 82 | Gy220 | temperate |
| 83 | Gy237 | temperate |
| 84 | Gy246 | temperate |
| 85 | Gy923 | temperate |
| 86 | Gy462 | temperate |

| | | |
|---|---|---|
| 87 | Ry713 | temperate |
| 88 | Sy999 | temperate |
| 89 | Sy1052 | temperate |
| 90 | Sy1077 | temperate |
| 91 | Sy3073 | temperate |
| 92 | Ye478 | temperate |
| 93 | Mo17 | temperate |
| 94 | Si444 | temperate |
| 95 | Ye52106 | temperate |
| 96 | 7884-4Ht | temperate |
| 97 | Shen5003 | temperate |
| 98 | U8112 | temperate |
| 99 | Zheng653 | temperate |
| 100 | Qi319 | tropical/subtropical |
| 101 | Zong31 | temperate |
| 102 | Dong46 | temperate |
| 103 | Ji846 | temperate |
| 104 | K10 | temperate |
| 105 | LK11 | temperate |
| 106 | Hu803 | temperate |
| 107 | P178 | tropical/subtropical |
| 108 | chuan48-2 | temperate |
| 109 | Tian77 | temperate |
| 110 | Ji853 | temperate |
| 111 | Tie7922 | temperate |
| 112 | Ye8001 | temperate |
| 113 | Wu109 | temperate |
| 114 | Lv28 | temperate |
| 115 | 812 | temperate |
| 116 | J4112 | temperate |
| 117 | Nan21-3 | tropical/subtropical |
| 118 | Qi205 | temperate |
| 119 | Shen137 | tropical/subtropical |
| 120 | Dan599 | tropical/subtropical |
| 121 | MO113 | temperate |
| 122 | K22 | temperate |
| 123 | Xi502 | temperate |
| 124 | WH413 | temperate |
| 125 | S37 | tropical/subtropical |
| 126 | Lx9801 | temperate |
| 127 | Dan598 | temperate |
| 128 | 4F1 | temperate |
| 129 | K14 | temperate |
| 130 | Zheng58 | temperate |

| | | |
|---|---|---|
| 131 | BS16 | temperate |
| 132 | BGY | temperate |
| 133 | SW92E114 | temperate |
| 134 | HB | temperate |
| 135 | DSB | temperate |
| 136 | WMR | temperate |
| 137 | MN | temperate |
| 138 | 04K5702 | temperate |
| 139 | 04K5672 | temperate |
| 140 | Xun971 | temperate |
| 141 | D047 | temperate |
| 142 | 1323 | temperate |
| 143 | 384-2 | temperate |
| 144 | Z2018F | temperate |
| 145 | M165 | temperate |
| 146 | 05WN230 | temperate |
| 147 | 975-12 | temperate |
| 148 | JY01 | temperate |
| 149 | LY042 | temperate |
| 150 | FCD0602 | temperate |
| 151 | CF3 | temperate |
| 152 | TT16 | temperate |
| 153 | DH3732 | temperate |
| 154 | Dan4245 | temperate |
| 155 | TY1 | temperate |
| 156 | TY2 | temperate |
| 157 | TY3 | temperate |
| 158 | TY4 | temperate |
| 159 | TY5 | temperate |
| 160 | TY6 | temperate |
| 161 | TY7 | temperate |
| 162 | TY8 | temperate |
| 163 | TY10 | temperate |
| 164 | TY11 | temperate |
| 165 | B110 | temperate |
| 166 | B111 | temperate |
| 167 | IRF314 | temperate |
| 168 | 3411 | temperate |
| 169 | 5213 | temperate |
| 170 | 501 | temperate |
| 171 | W138 | temperate |
| 172 | L3180 | temperate |
| 173 | 9642 | temperate |
| 174 | 177 | temperate |

| | | |
|---|---|---|
| 175 | 238 | temperate |
| 176 | Zheng35 | temperate |
| 177 | 4019 | temperate |
| 178 | ZZ03 | temperate |
| 179 | Zhong69 | temperate |
| 180 | JH59 | temperate |
| 181 | ZZ01 | temperate |
| 182 | Dan3130 | temperate |
| 183 | Liao159 | temperate |
| 184 | Dan360 | temperate |
| 185 | 1462 | temperate |
| 186 | JH96C | temperate |
| 187 | 268 | temperate |
| 188 | B151 | temperate |
| 189 | Liao5114 | temperate |
| 190 | D863F | temperate |
| 191 | 835b | temperate |
| 192 | Zheng28 | temperate |
| 193 | Zheng29 | temperate |
| 194 | EN25 | temperate |
| 195 | LXN | temperate |
| 196 | DH29 | temperate |
| 197 | 526018 | temperate |
| 198 | ES40 | temperate |
| 199 | R08 | temperate |
| 200 | GEMS3 | temperate |
| 201 | GEMS5 | temperate |
| 202 | GEMS6 | temperate |
| 203 | GEMS10 | temperate |
| 204 | GEMS11 | temperate |
| 205 | GEMS12 | temperate |
| 206 | GEMS13 | temperate |
| 207 | GEMS14 | temperate |
| 208 | GEMS15 | temperate |
| 209 | GEMS17 | temperate |
| 210 | GEMS19 | temperate |
| 211 | GEMS21 | temperate |
| 212 | GEMS25 | temperate |
| 213 | GEMS27 | temperate |
| 214 | GEMS28 | temperate |
| 215 | GEMS29 | temperate |
| 216 | GEMS30 | temperate |
| 217 | GEMS31 | temperate |
| 218 | GEMS32 | temperate |

| | | |
|---|---|---|
| 219 | GEMS36 | temperate |
| 220 | GEMS39 | temperate |
| 221 | GEMS42 | temperate |
| 222 | GEMS44 | temperate |
| 223 | GEMS48 | temperate |
| 224 | GEMS49 | temperate |
| 225 | GEMS51 | temperate |
| 226 | GEMS53 | temperate |
| 227 | GEMS55 | temperate |
| 228 | GEMS58 | temperate |
| 229 | GEMS61 | temperate |
| 230 | GEMS63 | temperate |

Table S2. The Hg concentration in maize tissues.

| Location | The materials | | Kernel (μg kg$^{-1}$) | Axis (μg kg$^{-1}$) | Stem (μg kg$^{-1}$) | Bract (μg kg$^{-1}$) | Leave (μg kg$^{-1}$) |
|---|---|---|---|---|---|---|---|
| Xixian | 177 | 1 | 0.59 | 1.95 | 4.61 | 8.31 | 26.66 |
| | | 2 | 1.02 | 2.27 | 5.09 | 8.80 | 24.02 |
| | | 3 | 1.23 | 1.96 | 6.35 | 8.56 | 26.09 |
| | 238 | 1 | 0.70 | 1.41 | 2.63 | 6.27 | 32.32 |
| | | 2 | 1.43 | 1.83 | 4.11 | 6.62 | 30.72 |
| | | 3 | 1.06 | 2.37 | 2.62 | 6.59 | 32.27 |
| | 268 | 1 | 0.58 | 1.84 | 4.97 | 7.75 | 25.00 |
| | | 2 | 0.47 | 2.97 | 6.36 | 8.44 | 26.50 |
| | | 3 | 0.53 | 2.11 | 5.21 | 8.10 | 27.93 |
| | 1462 | 1 | 0.70 | 1.74 | 4.71 | 5.49 | 19.72 |
| | | 2 | 1.04 | 2.07 | 6.06 | 5.66 | 19.16 |
| | | 3 | 1.02 | 2.51 | 6.43 | 5.57 | 20.19 |
| | 4019 | 1 | 1.42 | 2.23 | 6.03 | 7.47 | 21.12 |
| | | 2 | 1.56 | 2.19 | 5.24 | 6.72 | 19.42 |
| | | 3 | 0.89 | 3.71 | 6.39 | 7.09 | 19.52 |
| | 5213 | 1 | 0.53 | 6.34 | 4.36 | 4.71 | 22.24 |
| | | 2 | 0.42 | 4.75 | 3.87 | 5.11 | 20.65 |
| | | 3 | 0.62 | 7.04 | 3.67 | 5.66 | 22.19 |
| | 9642 | 1 | 0.65 | 2.11 | 5.04 | 4.91 | 22.75 |
| | | 2 | 0.34 | 2.38 | 4.03 | 5.33 | 21.16 |
| | | 3 | 0.46 | 2.25 | 4.69 | 5.87 | 22.70 |
| | 526018 | 1 | 0.96 | 1.79 | 4.02 | 5.46 | 24.41 |
| | | 2 | 1.52 | 2.08 | 3.58 | 5.46 | 24.40 |
| | | 3 | 1.12 | 2.09 | 3.50 | 5.46 | 24.40 |
| | 05WN230 | 1 | 1.16 | 1.16 | 6.45 | 5.28 | 27.77 |
| | | 2 | 0.84 | 2.05 | 6.50 | 6.18 | 27.50 |
| | | 3 | 0.70 | 2.36 | 7.08 | 5.73 | 27.64 |
| | 4F1 | 1 | 1.05 | 3.10 | 4.94 | 5.33 | 27.09 |
| | | 2 | 0.48 | 4.26 | 6.02 | 5.52 | 25.65 |
| | | 3 | 0.47 | 4.43 | 6.98 | 4.67 | 27.87 |
| | 7884-4HT | 1 | 1.07 | 3.32 | 6.75 | 7.23 | 25.12 |
| | | 2 | 0.51 | 4.76 | 7.21 | 6.94 | 23.19 |
| | | 3 | 0.49 | 5.54 | 7.28 | 7.08 | 24.90 |
| | 835B | 1 | 0.77 | 4.10 | 5.27 | 4.26 | 26.50 |
| | | 2 | 0.66 | 3.83 | 6.07 | 4.83 | 25.00 |
| | | 3 | 1.17 | 3.81 | 6.12 | 3.80 | 27.93 |
| | 975-12 | 1 | 1.56 | 3.15 | 9.06 | 8.24 | 25.66 |
| | | 2 | 0.95 | 3.51 | 8.27 | 7.00 | 23.95 |
| | | 3 | 1.56 | 2.88 | 9.12 | 6.87 | 24.05 |
| | B110 | 1 | 0.72 | 3.84 | 3.55 | 5.71 | 34.87 |
| | | 2 | 0.74 | 5.28 | 3.41 | 7.09 | 32.00 |

| | | | | | | |
|---|---|---|---|---|---|---|
| | 3 | 1.03 | 3.96 | 3.48 | 5.65 | 34.93 |
| B111 | 1 | 1.52 | 2.66 | 5.08 | 5.68 | 32.51 |
| | 2 | 0.68 | 4.02 | 5.24 | 6.50 | 30.57 |
| | 3 | 1.68 | 3.34 | 5.16 | 5.34 | 33.79 |
| B151 | 1 | 1.05 | 3.91 | 4.19 | 7.16 | 27.50 |
| | 2 | 1.06 | 4.89 | 4.40 | 7.29 | 24.68 |
| | 3 | 1.36 | 4.25 | 5.05 | 7.23 | 28.34 |
| BS16 | 1 | 0.81 | 1.69 | 5.50 | 6.01 | 30.09 |
| | 2 | 1.08 | 2.18 | 7.20 | 6.02 | 28.87 |
| | 3 | 1.09 | 1.63 | 7.85 | 5.26 | 30.23 |
| BY4839 | 1 | 1.78 | 3.73 | 4.90 | 7.72 | 35.83 |
| | 2 | 0.93 | 3.75 | 6.03 | 6.98 | 34.36 |
| | 3 | 0.91 | 3.74 | 6.52 | 8.10 | 35.84 |
| BY804 | 1 | 0.75 | 2.46 | 3.00 | 5.35 | 26.24 |
| | 2 | 0.43 | 2.88 | 3.05 | 4.97 | 24.07 |
| | 3 | 0.74 | 2.67 | 3.62 | 5.09 | 25.91 |
| BY807 | 1 | 0.65 | 2.30 | 6.34 | 6.06 | 25.31 |
| | 2 | 0.38 | 2.69 | 7.00 | 6.00 | 24.91 |
| | 3 | 0.28 | 2.49 | 6.37 | 6.03 | 25.11 |
| BY809 | 1 | 0.82 | 4.87 | 4.09 | 6.15 | 25.49 |
| | 2 | 0.56 | 6.19 | 3.67 | 7.10 | 24.41 |
| | 3 | 0.33 | 4.93 | 4.18 | 5.88 | 25.70 |
| BY813 | 1 | 1.57 | 2.81 | 5.07 | 6.00 | 35.31 |
| | 2 | 1.03 | 2.92 | 6.07 | 6.25 | 30.84 |
| | 3 | 1.30 | 2.86 | 4.97 | 6.88 | 34.57 |
| BY815 | 1 | 1.20 | 3.59 | 6.03 | 9.60 | 22.60 |
| | 2 | 0.90 | 3.31 | 5.58 | 9.97 | 22.60 |
| | 3 | 0.93 | 3.45 | 5.96 | 9.79 | 24.10 |
| BY855 | 1 | 1.71 | 3.29 | 5.84 | 5.08 | 25.24 |
| | 2 | 0.95 | 3.59 | 6.02 | 5.65 | 24.26 |
| | 3 | 0.88 | 3.59 | 6.53 | 5.36 | 24.00 |
| CHUAN48-2 | 1 | 0.58 | 3.32 | 4.42 | 4.42 | 22.10 |
| | 2 | 0.26 | 2.69 | 4.28 | 5.63 | 22.27 |
| | 3 | 0.57 | 3.30 | 4.35 | 4.28 | 22.18 |
| CIMBL1 | 1 | 1.05 | 1.57 | 4.09 | 6.40 | 22.98 |
| | 2 | 0.44 | 2.21 | 3.06 | 6.96 | 20.28 |
| | 3 | 0.45 | 3.39 | 4.02 | 7.43 | 21.63 |
| CIMBL105 | 1 | 0.19 | 4.25 | 3.36 | 4.85 | 24.56 |
| | 2 | 0.56 | 5.07 | 3.35 | 5.26 | 21.35 |
| | 3 | 0.23 | 4.21 | 3.35 | 5.20 | 24.45 |
| CIMBL106 | 1 | 0.69 | 1.22 | 6.06 | 3.28 | 29.65 |
| | 2 | 0.77 | 1.36 | 5.07 | 3.58 | 27.09 |
| | 3 | 0.73 | 1.29 | 5.71 | 3.70 | 29.12 |
| CIMBL109 | 1 | 0.75 | 3.15 | 6.63 | 7.23 | 32.14 |

| | | | | | | |
|---|---|---|---|---|---|---|
| | 2 | 0.62 | 3.09 | 5.36 | 6.75 | 30.11 |
| | 3 | 0.63 | 3.12 | 6.29 | 7.74 | 32.62 |
| CIMBL11 | 1 | 0.71 | 1.87 | 4.68 | 4.18 | 17.86 |
| | 2 | 0.56 | 2.58 | 6.43 | 4.34 | 17.57 |
| | 3 | 0.56 | 2.82 | 6.31 | 4.26 | 17.72 |
| CIMBL12 | 1 | 1.08 | 3.08 | 4.21 | 7.03 | 25.66 |
| | 2 | 0.82 | 3.34 | 3.75 | 6.76 | 22.65 |
| | 3 | 1.04 | 3.21 | 4.28 | 7.04 | 25.66 |
| CIMBL120 | 1 | 0.69 | 4.89 | 5.28 | 5.39 | 31.42 |
| | 2 | 1.02 | 6.47 | 4.40 | 5.76 | 29.02 |
| | 3 | 0.71 | 5.08 | 4.99 | 5.35 | 30.97 |
| CIMBL123 | 1 | 0.85 | 2.50 | 4.03 | 5.94 | 24.89 |
| | 2 | 0.54 | 2.40 | 5.07 | 6.00 | 22.65 |
| | 3 | 0.39 | 3.95 | 4.70 | 5.82 | 23.02 |
| CIMBL124 | 1 | 1.57 | 2.78 | 4.94 | 5.56 | 27.73 |
| | 2 | 0.85 | 2.90 | 7.06 | 5.57 | 24.96 |
| | 3 | 1.36 | 2.84 | 5.85 | 5.57 | 27.84 |
| CIMBL127 | 1 | 0.81 | 1.82 | 4.37 | 5.74 | 27.27 |
| | 2 | 0.35 | 1.94 | 4.13 | 5.80 | 26.20 |
| | 3 | 0.47 | 1.88 | 4.25 | 5.62 | 28.99 |
| CIMBL133 | 1 | 1.23 | 2.82 | 3.04 | 6.01 | 15.74 |
| | 2 | 1.28 | 2.39 | 1.88 | 6.04 | 15.33 |
| | 3 | 0.96 | 3.35 | 1.86 | 6.02 | 17.04 |
| CIMBL140 | 1 | 0.71 | 5.65 | 2.57 | 8.79 | 31.76 |
| | 2 | 1.07 | 5.69 | 5.07 | 9.29 | 30.29 |
| | 3 | 1.04 | 5.67 | 4.72 | 9.04 | 31.77 |
| CIMBL142 | 1 | 0.51 | 2.39 | 3.19 | 4.76 | 26.93 |
| | 2 | 0.43 | 2.77 | 1.86 | 5.77 | 26.87 |
| | 3 | 0.30 | 2.58 | 1.02 | 6.01 | 26.90 |
| CIMBL143 | 1 | 1.01 | 2.66 | 4.70 | 4.76 | 23.59 |
| | 2 | 0.51 | 2.99 | 4.08 | 4.41 | 23.59 |
| | 3 | 0.95 | 2.82 | 4.84 | 3.84 | 23.59 |
| CIMBL144 | 1 | 0.54 | 3.05 | 4.13 | 5.54 | 25.56 |
| | 2 | 0.59 | 2.90 | 2.98 | 5.26 | 23.53 |
| | 3 | 0.77 | 2.83 | 3.41 | 4.65 | 26.05 |
| CIMBL150 | 1 | 1.03 | 3.40 | 3.75 | 2.56 | 25.72 |
| | 2 | 2.79 | 3.24 | 3.64 | 3.03 | 25.23 |
| | 3 | 1.91 | 3.32 | 3.99 | 2.05 | 25.80 |
| CIMBL151 | 1 | 1.10 | 2.51 | 4.04 | 6.04 | 23.89 |
| | 2 | 1.83 | 2.77 | 3.33 | 6.73 | 24.01 |
| | 3 | 1.16 | 2.64 | 4.29 | 6.98 | 23.95 |
| CIMBL153 | 1 | 0.65 | 2.36 | 4.07 | 5.32 | 33.73 |
| | 2 | 0.76 | 2.53 | 3.05 | 5.41 | 31.61 |
| | 3 | 0.70 | 2.59 | 3.71 | 4.99 | 33.42 |

| | | | | | | |
|---|---|---|---|---|---|---|
| CIMBL157 | 1 | 0.86 | 3.24 | 1.86 | 5.75 | 23.22 |
| | 2 | 1.22 | 4.03 | 3.27 | 5.74 | 21.09 |
| | 3 | 0.70 | 3.33 | 3.46 | 5.00 | 22.91 |
| CIMBL2 | 1 | 2.69 | 2.00 | 7.43 | 10.71 | 29.03 |
| | 2 | 1.24 | 2.04 | 8.54 | 10.96 | 26.64 |
| | 3 | 1.36 | 1.57 | 7.99 | 10.09 | 28.58 |
| CIMBL21 | 1 | 1.68 | 1.90 | 4.06 | 5.04 | 31.84 |
| | 2 | 0.99 | 2.52 | 3.47 | 5.04 | 27.55 |
| | 3 | 1.80 | 2.36 | 4.51 | 5.49 | 31.20 |
| CIMBL23 | 1 | 0.79 | 1.78 | 5.29 | 4.45 | 18.75 |
| | 2 | 0.66 | 2.62 | 4.95 | 4.71 | 17.91 |
| | 3 | 0.72 | 2.35 | 5.72 | 3.98 | 19.08 |
| CIMBL32 | 1 | 0.73 | 2.48 | 3.05 | 3.71 | 27.72 |
| | 2 | 0.76 | 2.70 | 1.84 | 2.90 | 25.98 |
| | 3 | 1.02 | 2.59 | 2.60 | 3.01 | 28.35 |
| CIMBL38 | 1 | 0.94 | 2.08 | 5.00 | 8.08 | 30.48 |
| | 2 | 0.98 | 1.67 | 4.44 | 7.80 | 30.18 |
| | 3 | 0.96 | 2.18 | 5.62 | 7.19 | 30.33 |
| CIMBL42 | 1 | 0.48 | 2.01 | 8.53 | 9.51 | 23.56 |
| | 2 | 0.95 | 1.71 | 10.06 | 9.21 | 22.96 |
| | 3 | 0.57 | 2.16 | 10.64 | 8.84 | 23.26 |
| CIMBL48 | 1 | 0.14 | 2.94 | 5.84 | 6.06 | 36.92 |
| | 2 | 0.47 | 3.81 | 5.10 | 6.51 | 35.14 |
| | 3 | 0.31 | 3.52 | 5.47 | 5.76 | 36.78 |
| CIMBL49 | 1 | 1.03 | 1.97 | 4.11 | 5.99 | 30.48 |
| | 2 | 0.54 | 2.10 | 2.47 | 6.03 | 28.55 |
| | 3 | 0.64 | 2.18 | 2.69 | 6.53 | 30.27 |
| CIMBL58 | 1 | 0.59 | 2.88 | 4.00 | 4.19 | 16.43 |
| | 2 | 0.70 | 3.31 | 3.36 | 3.61 | 15.08 |
| | 3 | 0.50 | 3.54 | 3.83 | 4.48 | 17.25 |
| CIMBL59 | 1 | 1.04 | 2.06 | 3.49 | 4.28 | 37.22 |
| | 2 | 0.82 | 2.11 | 3.34 | 5.01 | 33.55 |
| | 3 | 0.48 | 2.09 | 4.16 | 4.57 | 34.63 |
| CIMBL60 | 1 | 0.89 | 3.47 | 5.25 | 5.00 | 18.28 |
| | 2 | 0.42 | 3.44 | 4.60 | 4.67 | 19.12 |
| | 3 | 0.50 | 3.45 | 5.37 | 4.24 | 18.88 |
| CIMBL75 | 1 | 1.22 | 2.26 | 3.41 | 5.03 | 29.40 |
| | 2 | 2.07 | 2.12 | 5.08 | 4.50 | 26.21 |
| | 3 | 2.55 | 2.19 | 4.54 | 5.55 | 28.55 |
| CIMBL77 | 1 | 0.43 | 2.99 | 3.54 | 3.32 | 28.65 |
| | 2 | 0.47 | 4.06 | 3.70 | 3.54 | 26.96 |
| | 3 | 0.75 | 3.53 | 4.07 | 4.11 | 28.56 |
| CIMBL84 | 1 | 1.00 | 1.71 | 4.09 | 4.65 | 21.25 |
| | 2 | 0.60 | 2.08 | 3.94 | 5.01 | 20.46 |

| | | | | | | |
|---|---|---|---|---|---|---|
| | 3 | 0.50 | 1.59 | 4.47 | 4.98 | 20.10 |
| CIMBL86 | 1 | 0.83 | 2.45 | 5.79 | 4.78 | 22.51 |
| | 2 | 0.59 | 2.69 | 6.06 | 5.01 | 21.58 |
| | 3 | 0.86 | 2.57 | 6.23 | 5.19 | 22.74 |
| CIMBL87 | 1 | 1.61 | 2.56 | 2.91 | 5.33 | 23.89 |
| | 2 | 0.90 | 2.00 | 3.76 | 5.11 | 20.54 |
| | 3 | 1.11 | 1.98 | 3.63 | 5.75 | 23.71 |
| CIMBL88 | 1 | 1.60 | 2.01 | 3.35 | 6.74 | 24.36 |
| | 2 | 2.96 | 3.36 | 1.54 | 6.29 | 23.38 |
| | 3 | 2.28 | 4.18 | 1.69 | 6.74 | 25.37 |
| CIMBL91 | 1 | 1.06 | 3.77 | 7.06 | 7.89 | 23.86 |
| | 2 | 0.50 | 4.40 | 6.41 | 7.42 | 25.77 |
| | 3 | 0.63 | 3.33 | 6.20 | 8.03 | 24.06 |
| CIMBL92 | 1 | 0.76 | 2.00 | 3.41 | 2.84 | 27.37 |
| | 2 | 1.02 | 2.46 | 4.36 | 3.54 | 27.44 |
| | 3 | 1.19 | 2.23 | 4.63 | 3.64 | 28.90 |
| CIMBL93 | 1 | 1.21 | 3.05 | 5.02 | 4.76 | 30.31 |
| | 2 | 0.64 | 3.61 | 4.38 | 4.95 | 27.64 |
| | 3 | 0.77 | 3.33 | 5.15 | 4.33 | 29.73 |
| CIMBL96 | 1 | 0.75 | 4.36 | 5.32 | 5.78 | 21.39 |
| | 2 | 0.77 | 4.47 | 4.39 | 6.15 | 20.57 |
| | 3 | 1.05 | 3.81 | 5.45 | 6.42 | 21.73 |
| CML115 | 1 | 1.54 | 3.13 | 6.08 | 5.75 | 32.97 |
| | 2 | 2.09 | 1.90 | 5.04 | 6.26 | 29.63 |
| | 3 | 2.11 | 2.22 | 4.81 | 6.53 | 32.80 |
| CML116 | 1 | 0.60 | 0.90 | 3.03 | 2.94 | 30.04 |
| | 2 | 0.21 | 2.04 | 2.30 | 3.52 | 29.98 |
| | 3 | 0.41 | 1.62 | 1.16 | 3.38 | 32.26 |
| CML118 | 1 | 0.84 | 3.55 | 5.36 | 7.09 | 31.46 |
| | 2 | 1.00 | 5.02 | 5.40 | 6.64 | 29.10 |
| | 3 | 0.77 | 4.59 | 5.38 | 7.09 | 31.78 |
| CML121 | 1 | 0.81 | 2.70 | 5.72 | 4.32 | 24.01 |
| | 2 | 0.69 | 3.05 | 6.16 | 4.50 | 23.83 |
| | 3 | 0.60 | 2.58 | 6.09 | 3.66 | 24.42 |
| CML122 | 1 | 1.20 | 1.90 | 6.12 | 3.11 | 30.67 |
| | 2 | 0.91 | 2.37 | 6.12 | 3.51 | 29.27 |
| | 3 | 1.35 | 2.58 | 6.12 | 3.53 | 31.47 |
| CML130 | 1 | 0.44 | 3.41 | 9.58 | 5.17 | 25.90 |
| | 2 | 0.47 | 2.80 | 9.16 | 6.12 | 25.51 |
| | 3 | 0.75 | 3.41 | 9.37 | 5.80 | 27.20 |
| CML139 | 1 | 0.90 | 2.30 | 5.06 | 3.96 | 30.18 |
| | 2 | 1.02 | 1.80 | 4.72 | 4.95 | 29.82 |
| | 3 | 1.11 | 2.20 | 5.94 | 4.30 | 30.75 |
| CML163 | 1 | 1.31 | 3.84 | 2.52 | 6.01 | 21.82 |

| | | | | | | |
|---|---|---|---|---|---|---|
| | 2 | 0.88 | 5.28 | 2.07 | 5.99 | 20.39 |
| | 3 | 0.81 | 4.86 | 1.99 | 5.82 | 21.86 |
| CML165 | 1 | 0.88 | 3.29 | 3.92 | 3.48 | 25.35 |
| | 2 | 0.86 | 5.36 | 5.47 | 3.93 | 23.77 |
| | 3 | 0.87 | 4.17 | 5.30 | 3.10 | 25.31 |
| CML170 | 1 | 1.04 | 1.74 | 5.09 | 4.52 | 32.73 |
| | 2 | 0.53 | 1.94 | 4.87 | 5.81 | 31.31 |
| | 3 | 0.49 | 1.84 | 5.43 | 5.91 | 32.77 |
| CML290 | 1 | 1.02 | 3.93 | 3.72 | 6.90 | 24.33 |
| | 2 | 0.51 | 4.30 | 5.06 | 6.91 | 23.46 |
| | 3 | 0.61 | 4.57 | 5.14 | 7.65 | 23.14 |
| CML304 | 1 | 1.22 | 3.56 | 5.25 | 4.18 | 31.20 |
| | 2 | 0.87 | 3.34 | 3.69 | 3.92 | 29.30 |
| | 3 | 0.75 | 3.45 | 4.02 | 3.30 | 31.00 |
| CML31 | 1 | 1.23 | 3.11 | 5.03 | 4.82 | 25.33 |
| | 2 | 0.35 | 2.86 | 5.10 | 5.00 | 22.75 |
| | 3 | 0.62 | 3.14 | 5.06 | 5.14 | 24.79 |
| CML325 | 1 | 1.55 | 3.74 | 4.05 | 4.86 | 31.58 |
| | 2 | 0.64 | 5.03 | 4.15 | 5.79 | 29.39 |
| | 3 | 0.95 | 5.13 | 5.15 | 5.62 | 31.24 |
| CML338 | 1 | 1.57 | 3.39 | 6.24 | 4.27 | 20.22 |
| | 2 | 1.33 | 3.87 | 5.04 | 4.64 | 19.73 |
| | 3 | 1.27 | 3.63 | 6.39 | 3.78 | 21.47 |
| CML423 | 1 | 1.10 | 3.59 | 6.23 | 4.77 | 30.78 |
| | 2 | 0.76 | 3.77 | 5.57 | 5.00 | 31.39 |
| | 3 | 1.08 | 3.68 | 6.20 | 4.41 | 33.33 |
| CML479 | 1 | 1.09 | 2.16 | 5.11 | 2.96 | 26.26 |
| | 2 | 0.72 | 4.09 | 4.92 | 3.26 | 23.13 |
| | 3 | 0.80 | 3.73 | 4.42 | 2.44 | 25.45 |
| D863F | 1 | 0.39 | 3.58 | 7.00 | 6.49 | 25.08 |
| | 2 | 0.12 | 5.45 | 6.80 | 5.63 | 25.01 |
| | 3 | 0.11 | 4.82 | 5.40 | 5.61 | 25.05 |
| DAN3130 | 1 | 1.42 | 2.25 | 4.30 | 5.30 | 23.55 |
| | 2 | 0.98 | 3.05 | 6.44 | 6.11 | 21.69 |
| | 3 | 1.51 | 2.80 | 5.97 | 6.01 | 24.12 |
| DAN360 | 1 | 3.07 | 1.78 | 4.97 | 12.63 | 21.70 |
| | 2 | 1.48 | 1.74 | 4.41 | 12.26 | 22.44 |
| | 3 | 2.38 | 1.76 | 4.69 | 13.20 | 22.82 |
| DAN4245 | 1 | 0.64 | 3.22 | 5.08 | 9.64 | 25.36 |
| | 2 | 1.02 | 2.78 | 4.94 | 10.54 | 23.46 |
| | 3 | 0.98 | 3.30 | 6.06 | 10.24 | 25.91 |
| DAN599 | 1 | 0.28 | 1.99 | 4.06 | 5.37 | 24.17 |
| | 2 | 0.36 | 1.80 | 3.70 | 5.73 | 21.46 |
| | 3 | 0.48 | 1.75 | 4.18 | 4.95 | 23.57 |

| | | | | | | |
|---|---|---|---|---|---|---|
| DH3732 | 1 | 0.91 | 5.97 | 3.73 | 4.81 | 23.02 |
| | 2 | 1.12 | 7.02 | 4.11 | 5.01 | 23.74 |
| | 3 | 0.95 | 5.00 | 4.22 | 5.29 | 26.38 |
| DONG46 | 1 | 1.14 | 1.78 | 5.80 | 3.72 | 43.64 |
| | 2 | 0.94 | 2.05 | 7.38 | 4.06 | 40.71 |
| | 3 | 0.74 | 2.21 | 5.09 | 3.29 | 42.93 |
| EN25 | 1 | 1.66 | 2.56 | 4.83 | 5.54 | 25.00 |
| | 2 | 1.00 | 3.18 | 5.03 | 5.51 | 26.00 |
| | 3 | 1.09 | 3.47 | 5.53 | 4.77 | 28.47 |
| ES40 | 1 | 1.11 | 2.56 | 2.13 | 4.06 | 21.77 |
| | 2 | 1.25 | 3.21 | 2.60 | 3.63 | 19.99 |
| | 3 | 1.00 | 3.49 | 2.67 | 3.17 | 21.63 |
| FCD0602 | 1 | 1.21 | 1.00 | 5.03 | 5.05 | 25.06 |
| | 2 | 2.21 | 2.53 | 4.80 | 6.18 | 22.06 |
| | 3 | 1.50 | 2.07 | 4.92 | 5.84 | 25.81 |
| GEMS10 | 1 | 1.07 | 1.37 | 4.37 | 15.15 | 26.94 |
| | 2 | 0.25 | 2.76 | 4.35 | 10.90 | 23.01 |
| | 3 | 0.51 | 2.37 | 4.36 | 14.15 | 25.72 |
| GEMS11 | 1 | 1.20 | 3.90 | 5.44 | 6.91 | 25.69 |
| | 2 | 0.78 | 5.21 | 6.33 | 6.99 | 25.31 |
| | 3 | 0.69 | 5.61 | 5.56 | 6.65 | 25.87 |
| GEMS13 | 1 | 0.70 | 1.95 | 3.53 | 3.88 | 29.90 |
| | 2 | 0.72 | 3.02 | 3.52 | 4.05 | 29.60 |
| | 3 | 0.71 | 2.18 | 3.52 | 3.22 | 29.00 |
| GEMS14 | 1 | 1.19 | 2.98 | 2.57 | 4.73 | 29.86 |
| | 2 | 2.00 | 3.49 | 4.30 | 5.85 | 27.28 |
| | 3 | 1.79 | 2.64 | 3.29 | 6.04 | 28.57 |
| GEMS15 | 1 | 1.65 | 2.02 | 3.66 | 6.80 | 25.33 |
| | 2 | 1.37 | 2.30 | 4.64 | 6.75 | 22.21 |
| | 3 | 1.22 | 2.16 | 3.85 | 6.77 | 24.52 |
| GEMS17 | 1 | 0.92 | 2.68 | 4.35 | 4.75 | 25.01 |
| | 2 | 1.07 | 5.03 | 4.09 | 5.34 | 23.74 |
| | 3 | 1.29 | 4.90 | 4.22 | 5.49 | 25.13 |
| GEMS19 | 1 | 1.04 | 2.28 | 5.39 | 6.50 | 28.23 |
| | 2 | 0.93 | 2.37 | 5.12 | 6.17 | 26.20 |
| | 3 | 0.68 | 2.32 | 6.01 | 6.63 | 27.96 |
| GEMS21 | 1 | 0.18 | 4.51 | 4.85 | 5.51 | 29.60 |
| | 2 | 0.79 | 3.91 | 3.82 | 6.55 | 28.12 |
| | 3 | 0.65 | 4.36 | 4.05 | 6.56 | 30.36 |
| GEMS25 | 1 | 0.80 | 2.30 | 3.51 | 5.07 | 29.43 |
| | 2 | 1.01 | 3.08 | 3.43 | 5.44 | 27.02 |
| | 3 | 1.11 | 2.84 | 3.47 | 6.01 | 29.72 |
| GEMS28 | 1 | 1.65 | 4.15 | 7.60 | 4.86 | 28.96 |
| | 2 | 0.78 | 5.53 | 8.12 | 5.22 | 26.84 |

| | | | | | | |
|---|---|---|---|---|---|---|
| | 3 | 1.51 | 5.29 | 8.30 | 5.79 | 29.40 |
| GEMS29 | 1 | 0.93 | 1.91 | 3.26 | 4.69 | 29.08 |
| | 2 | 1.32 | 3.18 | 3.39 | 4.61 | 28.79 |
| | 3 | 1.38 | 2.25 | 3.33 | 5.40 | 29.69 |
| GEMS3 | 1 | 1.37 | 1.97 | 5.06 | 5.10 | 21.45 |
| | 2 | 1.93 | 3.04 | 4.30 | 5.98 | 21.14 |
| | 3 | 2.55 | 2.35 | 5.80 | 4.79 | 22.04 |
| GEMS30 | 1 | 1.38 | 2.05 | 5.00 | 5.09 | 27.68 |
| | 2 | 1.53 | 2.24 | 2.87 | 4.66 | 26.86 |
| | 3 | 2.06 | 2.74 | 3.63 | 5.62 | 26.52 |
| GEMS31 | 1 | 1.52 | 2.08 | 3.55 | 3.86 | 32.07 |
| | 2 | 0.88 | 1.72 | 4.24 | 4.20 | 28.35 |
| | 3 | 1.54 | 2.05 | 4.35 | 3.28 | 31.71 |
| GEMS32 | 1 | 2.56 | 2.57 | 5.30 | 6.54 | 25.54 |
| | 2 | 2.01 | 2.04 | 3.81 | 5.96 | 21.82 |
| | 3 | 1.73 | 2.46 | 5.15 | 6.93 | 24.43 |
| GEMS36 | 1 | 1.88 | 1.81 | 16.82 | 7.77 | 25.08 |
| | 2 | 0.87 | 2.12 | 12.57 | 8.33 | 21.32 |
| | 3 | 1.08 | 2.12 | 13.79 | 8.58 | 23.95 |
| GEMS39 | 1 | 1.27 | 2.94 | 5.14 | 6.69 | 32.90 |
| | 2 | 1.28 | 3.43 | 4.46 | 7.34 | 29.71 |
| | 3 | 1.28 | 3.49 | 4.35 | 5.51 | 32.80 |
| GEMS42 | 1 | 0.27 | 3.55 | 3.73 | 5.31 | 27.46 |
| | 2 | 0.73 | 4.15 | 5.01 | 5.33 | 26.73 |
| | 3 | 0.80 | 3.70 | 5.40 | 4.57 | 27.85 |
| GEMS44 | 1 | 1.00 | 3.59 | 5.26 | 8.56 | 26.68 |
| | 2 | 0.53 | 5.33 | 5.83 | 9.16 | 25.15 |
| | 3 | 1.04 | 4.91 | 5.54 | 9.61 | 27.42 |
| GEMS48 | 1 | 1.38 | 2.33 | 5.07 | 5.41 | 23.25 |
| | 2 | 1.73 | 2.43 | 4.04 | 5.54 | 22.50 |
| | 3 | 1.86 | 2.38 | 4.85 | 5.93 | 23.63 |
| GEMS49 | 1 | 0.98 | 3.94 | 3.67 | 6.98 | 24.14 |
| | 2 | 0.68 | 6.02 | 2.85 | 6.70 | 24.03 |
| | 3 | 1.13 | 5.13 | 2.96 | 7.59 | 24.09 |
| GEMS5 | 1 | 1.30 | 2.93 | 4.29 | 6.91 | 27.72 |
| | 2 | 1.44 | 2.43 | 3.85 | 7.93 | 25.09 |
| | 3 | 1.77 | 2.38 | 4.37 | 7.95 | 27.15 |
| GEMS51 | 1 | 0.88 | 2.82 | 9.01 | 7.53 | 21.85 |
| | 2 | 0.68 | 3.12 | 7.01 | 7.44 | 21.35 |
| | 3 | 1.18 | 3.12 | 8.01 | 8.01 | 21.75 |
| GEMS55 | 1 | 3.34 | 4.24 | 5.25 | 6.00 | 27.02 |
| | 2 | 2.44 | 4.12 | 3.80 | 6.04 | 24.80 |
| | 3 | 2.31 | 4.18 | 3.02 | 6.47 | 26.66 |
| GEMS58 | 1 | 0.77 | 2.31 | 4.16 | 4.51 | 28.13 |

| | | | | | | |
|---|---|---|---|---|---|---|
| | 2 | 0.66 | 2.26 | 5.01 | 4.46 | 26.98 |
| | 3 | 1.16 | 2.29 | 5.04 | 4.11 | 28.31 |
| GEMS6 | 1 | 1.14 | 3.47 | 6.51 | 7.54 | 20.01 |
| | 2 | 0.77 | 5.02 | 7.23 | 6.93 | 19.97 |
| | 3 | 0.82 | 5.60 | 7.12 | 7.24 | 19.01 |
| GEMS61 | 1 | 1.35 | 2.37 | 2.12 | 6.30 | 23.75 |
| | 2 | 2.01 | 3.24 | 1.78 | 6.58 | 21.74 |
| | 3 | 2.25 | 3.26 | 2.25 | 5.69 | 23.49 |
| GEMS63 | 1 | 0.92 | 2.52 | 5.24 | 5.82 | 22.29 |
| | 2 | 0.53 | 2.90 | 5.41 | 6.09 | 17.92 |
| | 3 | 0.88 | 2.71 | 5.32 | 5.20 | 21.60 |
| GY462 | 1 | 0.42 | 3.25 | 5.11 | 6.67 | 26.88 |
| | 2 | 0.78 | 2.76 | 6.25 | 5.18 | 24.19 |
| | 3 | 0.74 | 3.31 | 6.59 | 5.17 | 26.29 |
| GY923 | 1 | 0.22 | 2.14 | 2.94 | 6.23 | 27.99 |
| | 2 | 0.52 | 4.06 | 4.64 | 5.78 | 32.66 |
| | 3 | 0.22 | 3.85 | 4.09 | 6.83 | 29.83 |
| IRF314 | 1 | 1.08 | 3.92 | 5.05 | 8.96 | 31.28 |
| | 2 | 1.01 | 4.28 | 4.44 | 8.66 | 29.28 |
| | 3 | 1.04 | 4.85 | 4.31 | 9.56 | 31.78 |
| J4112 | 1 | 1.31 | 2.04 | 3.21 | 6.17 | 24.89 |
| | 2 | 0.86 | 2.65 | 3.06 | 5.54 | 21.63 |
| | 3 | 1.23 | 2.64 | 3.89 | 6.15 | 24.01 |
| JH59 | 1 | 0.26 | 4.21 | 4.56 | 5.69 | 32.41 |
| | 2 | 0.73 | 3.53 | 3.41 | 6.32 | 31.50 |
| | 3 | 0.50 | 3.72 | 4.31 | 5.26 | 32.74 |
| JH96C | 1 | 0.17 | 2.65 | 6.26 | 6.63 | 21.23 |
| | 2 | 0.44 | 2.64 | 6.43 | 7.25 | 21.25 |
| | 3 | 0.28 | 2.65 | 7.40 | 6.19 | 21.98 |
| JI846 | 1 | 0.97 | 2.70 | 1.32 | 4.00 | 29.84 |
| | 2 | 0.61 | 3.18 | 2.04 | 4.44 | 30.38 |
| | 3 | 0.97 | 3.69 | 2.43 | 3.53 | 29.66 |
| JI853 | 1 | 1.03 | 2.52 | 4.77 | 5.17 | 26.05 |
| | 2 | 0.64 | 2.05 | 4.28 | 6.14 | 26.01 |
| | 3 | 0.91 | 2.59 | 5.58 | 6.41 | 26.08 |
| JY01 | 1 | 1.06 | 2.24 | 4.13 | 7.41 | 21.35 |
| | 2 | 0.95 | 1.92 | 3.48 | 7.51 | 20.54 |
| | 3 | 1.00 | 2.83 | 4.57 | 8.21 | 21.69 |
| K10 | 1 | 1.38 | 2.73 | 6.65 | 5.63 | 26.89 |
| | 2 | 2.16 | 3.64 | 9.15 | 5.54 | 24.84 |
| | 3 | 1.42 | 3.48 | 8.95 | 6.03 | 26.62 |
| K14 | 1 | 1.96 | 2.66 | 4.12 | 4.36 | 36.22 |
| | 2 | 0.86 | 2.72 | 3.72 | 4.16 | 34.50 |
| | 3 | 0.96 | 2.69 | 4.40 | 5.01 | 36.12 |

| | | | | | | |
|---|---|---|---|---|---|---|
| K22 | 1 | 0.80 | 2.03 | 6.71 | 4.18 | 26.69 |
| | 2 | 1.00 | 2.85 | 7.13 | 5.12 | 25.94 |
| | 3 | 1.02 | 2.14 | 6.47 | 5.17 | 27.07 |
| L3180 | 1 | 1.06 | 3.87 | 2.13 | 6.89 | 35.31 |
| | 2 | 1.61 | 6.06 | 2.34 | 6.06 | 33.08 |
| | 3 | 0.73 | 5.27 | 2.83 | 6.77 | 34.95 |
| LIAO159 | 1 | 0.83 | 2.65 | 4.78 | 8.48 | 26.81 |
| | 2 | 0.56 | 2.77 | 5.09 | 9.30 | 26.99 |
| | 3 | 0.83 | 3.01 | 4.63 | 8.14 | 28.40 |
| LIAO5114 | 1 | 1.38 | 4.04 | 4.29 | 10.20 | 26.28 |
| | 2 | 0.62 | 3.81 | 6.22 | 10.36 | 24.07 |
| | 3 | 0.69 | 4.22 | 7.05 | 11.03 | 26.68 |
| LK11 | 1 | 1.10 | 2.01 | 3.05 | 4.84 | 34.61 |
| | 2 | 0.87 | 1.85 | 5.83 | 4.02 | 28.46 |
| | 3 | 1.14 | 2.23 | 2.94 | 4.96 | 25.12 |
| LV28 | 1 | 0.41 | 3.05 | 5.34 | 5.11 | 30.93 |
| | 2 | 0.42 | 2.61 | 3.89 | 7.28 | 29.73 |
| | 3 | 0.56 | 3.28 | 4.99 | 6.94 | 31.45 |
| LX9801 | 1 | 2.05 | 1.68 | 6.43 | 5.43 | 31.09 |
| | 2 | 0.95 | 3.07 | 6.38 | 6.38 | 28.75 |
| | 3 | 1.05 | 2.68 | 6.41 | 5.15 | 30.74 |
| LXN | 1 | 1.01 | 3.46 | 3.43 | 5.12 | 27.56 |
| | 2 | 0.71 | 4.72 | 5.28 | 5.83 | 25.08 |
| | 3 | 0.53 | 4.39 | 5.85 | 3.98 | 27.07 |
| LY042 | 1 | 0.73 | 0.85 | 10.55 | 3.94 | 26.70 |
| | 2 | 0.43 | 1.07 | 10.14 | 4.03 | 22.82 |
| | 3 | 0.67 | 1.11 | 11.84 | 4.21 | 25.51 |
| MO113 | 1 | 0.57 | 1.06 | 3.70 | 8.69 | 24.63 |
| | 2 | 0.65 | 2.69 | 3.50 | 9.26 | 24.04 |
| | 3 | 0.61 | 2.32 | 3.60 | 8.23 | 25.36 |
| MO17 | 1 | 1.35 | 2.85 | 4.51 | 7.10 | 36.42 |
| | 2 | 1.04 | 2.45 | 3.92 | 7.10 | 33.41 |
| | 3 | 2.10 | 2.50 | 4.51 | 8.60 | 37.20 |
| NAN21-3 | 1 | 2.12 | 1.85 | 4.65 | 6.88 | 24.06 |
| | 2 | 1.25 | 3.01 | 6.54 | 7.20 | 22.86 |
| | 3 | 2.09 | 2.58 | 6.20 | 7.49 | 24.37 |
| P178 | 1 | 0.45 | 3.39 | 11.83 | 5.47 | 32.77 |
| | 2 | 1.01 | 3.60 | 10.67 | 5.29 | 32.09 |
| | 3 | 0.58 | 3.50 | 12.75 | 4.63 | 34.68 |
| QI205 | 1 | 1.46 | 0.92 | 4.18 | 6.09 | 21.20 |
| | 2 | 0.84 | 1.80 | 4.32 | 5.58 | 20.96 |
| | 3 | 1.45 | 1.21 | 4.25 | 6.58 | 21.86 |
| RY713 | 1 | 0.71 | 2.24 | 4.25 | 5.46 | 19.02 |
| | 2 | 0.46 | 4.05 | 3.85 | 6.45 | 18.58 |

| | | | | | | |
|---|---|---|---|---|---|---|
| | 3 | 0.48 | 3.44 | 4.35 | 5.20 | 20.31 |
| S37 | 1 | 1.34 | 1.86 | 3.95 | 4.48 | 19.27 |
| | 2 | 0.92 | 2.78 | 4.15 | 5.14 | 17.97 |
| | 3 | 1.20 | 2.47 | 4.50 | 4.06 | 19.90 |
| SHEN5003 | 1 | 3.58 | 3.77 | 5.33 | 5.40 | 33.54 |
| | 2 | 3.27 | 3.29 | 7.13 | 5.02 | 30.83 |
| | 3 | 3.42 | 3.53 | 6.23 | 5.74 | 32.93 |
| SW92E114 | 1 | 0.99 | 1.94 | 4.57 | 6.48 | 32.58 |
| | 2 | 1.06 | 3.05 | 4.68 | 5.72 | 29.96 |
| | 3 | 1.02 | 2.33 | 4.63 | 6.85 | 32.00 |
| SY1052 | 1 | 1.00 | 2.16 | 3.59 | 6.37 | 23.52 |
| | 2 | 0.74 | 2.42 | 3.47 | 5.66 | 22.63 |
| | 3 | 1.02 | 2.41 | 3.53 | 6.77 | 23.88 |
| SY3073 | 1 | 0.54 | 3.84 | 3.06 | 6.00 | 22.96 |
| | 2 | 0.24 | 7.06 | 1.87 | 5.75 | 20.60 |
| | 3 | 0.39 | 6.35 | 2.61 | 6.18 | 22.53 |
| TIAN77 | 1 | 2.35 | 2.58 | 4.10 | 6.11 | 25.43 |
| | 2 | 1.22 | 3.01 | 6.01 | 6.25 | 23.89 |
| | 3 | 1.33 | 2.62 | 5.32 | 6.78 | 25.18 |
| TIE7922 | 1 | 1.77 | 4.42 | 4.39 | 3.33 | 24.45 |
| | 2 | 1.22 | 3.82 | 4.13 | 3.67 | 22.34 |
| | 3 | 1.72 | 3.94 | 4.26 | 3.95 | 24.14 |
| TY1 | 1 | 0.70 | 3.89 | 4.26 | 7.27 | 32.63 |
| | 2 | 0.56 | 3.76 | 4.03 | 7.17 | 31.70 |
| | 3 | 0.78 | 3.83 | 4.89 | 7.97 | 33.62 |
| TY11 | 1 | 1.01 | 4.07 | 3.72 | 4.70 | 30.08 |
| | 2 | 0.59 | 2.97 | 3.79 | 5.03 | 29.00 |
| | 3 | 0.45 | 3.37 | 3.75 | 5.24 | 30.29 |
| TY2 | 1 | 0.57 | 3.51 | 4.06 | 5.99 | 24.77 |
| | 2 | 0.44 | 3.62 | 3.43 | 6.05 | 24.08 |
| | 3 | 0.98 | 4.02 | 4.50 | 5.57 | 25.47 |
| TY3 | 1 | 0.57 | 4.91 | 7.02 | 3.16 | 11.82 |
| | 2 | 0.62 | 5.04 | 6.62 | 4.13 | 11.20 |
| | 3 | 0.73 | 4.68 | 6.37 | 4.02 | 13.76 |
| TY4 | 1 | 1.19 | 2.69 | 4.60 | 6.18 | 29.82 |
| | 2 | 0.87 | 3.02 | 5.29 | 6.09 | 30.46 |
| | 3 | 0.88 | 3.30 | 5.39 | 6.66 | 30.97 |
| TY5 | 1 | 1.67 | 2.00 | 3.71 | 5.43 | 27.54 |
| | 2 | 0.93 | 2.57 | 3.24 | 4.84 | 26.39 |
| | 3 | 1.00 | 2.58 | 3.78 | 5.44 | 28.46 |
| TY6 | 1 | 1.41 | 2.87 | 3.76 | 8.59 | 23.30 |
| | 2 | 0.95 | 3.04 | 3.36 | 8.93 | 22.40 |
| | 3 | 1.48 | 2.95 | 3.56 | 8.01 | 23.39 |
| U8112 | 1 | 0.85 | 3.64 | 4.62 | 4.82 | 27.52 |

| | | | | | | |
|---|---|---|---|---|---|---|
| | 2 | 0.78 | 6.02 | 5.34 | 5.14 | 25.60 |
| | 3 | 0.66 | 6.58 | 5.28 | 5.73 | 28.81 |
| W138 | 1 | 0.53 | 2.88 | 7.92 | 8.40 | 27.25 |
| | 2 | 1.03 | 3.23 | 7.19 | 8.19 | 25.69 |
| | 3 | 0.63 | 2.90 | 6.06 | 9.05 | 27.68 |
| WH413 | 1 | 1.25 | 3.01 | 3.15 | 5.08 | 24.09 |
| | 2 | 1.47 | 4.01 | 2.00 | 6.68 | 22.15 |
| | 3 | 1.96 | 3.81 | 3.62 | 6.63 | 23.87 |
| WU109 | 1 | 1.16 | 2.66 | 5.11 | 5.79 | 25.26 |
| | 2 | 1.12 | 2.30 | 3.62 | 5.68 | 22.96 |
| | 3 | 1.44 | 2.48 | 4.66 | 6.26 | 24.86 |
| XI502 | 1 | 0.58 | 2.39 | 3.38 | 6.61 | 22.01 |
| | 2 | 0.54 | 1.70 | 2.07 | 6.22 | 21.90 |
| | 3 | 0.83 | 1.90 | 3.17 | 7.17 | 22.62 |
| XUN971 | 1 | 0.62 | 3.76 | 6.72 | 4.94 | 29.96 |
| | 2 | 0.31 | 3.44 | 4.02 | 5.26 | 29.00 |
| | 3 | 0.46 | 3.60 | 6.87 | 4.72 | 30.23 |
| YE478 | 1 | 1.72 | 4.19 | 4.76 | 7.48 | 24.14 |
| | 2 | 1.21 | 3.46 | 3.00 | 6.65 | 20.71 |
| | 3 | 1.76 | 3.64 | 2.68 | 7.82 | 23.18 |
| YE52106 | 1 | 0.80 | 1.80 | 5.03 | 6.56 | 29.94 |
| | 2 | 0.81 | 2.72 | 4.26 | 6.43 | 27.92 |
| | 3 | 0.80 | 2.56 | 5.24 | 7.26 | 29.16 |
| YE8001 | 1 | 2.63 | 2.84 | 5.18 | 8.85 | 25.63 |
| | 2 | 3.02 | 2.23 | 4.87 | 9.35 | 25.21 |
| | 3 | 1.32 | 2.69 | 5.77 | 9.48 | 26.95 |
| Z2018F | 1 | 3.17 | 2.39 | 5.07 | 9.99 | 27.88 |
| | 2 | 3.44 | 1.97 | 4.03 | 8.95 | 25.36 |
| | 3 | 4.04 | 1.88 | 3.95 | 10.14 | 27.37 |
| ZHENG28 | 1 | 1.45 | 2.50 | 2.89 | 5.86 | 34.63 |
| | 2 | 2.08 | 2.76 | 4.02 | 5.82 | 32.37 |
| | 3 | 1.65 | 2.93 | 3.15 | 6.22 | 34.92 |
| ZHENG29 | 1 | 1.11 | 2.20 | 3.05 | 6.04 | 32.23 |
| | 2 | 1.07 | 2.24 | 1.88 | 5.63 | 28.72 |
| | 3 | 1.09 | 2.22 | 2.01 | 6.21 | 31.14 |
| ZHENG35 | 1 | 0.57 | 1.62 | 9.13 | 4.13 | 27.74 |
| | 2 | 0.59 | 0.80 | 9.05 | 5.12 | 25.90 |
| | 3 | 0.74 | 1.81 | 9.09 | 5.15 | 27.49 |
| ZHENG653 | 1 | 0.87 | 3.65 | 5.36 | 9.27 | 27.81 |
| | 2 | 1.00 | 3.81 | 5.06 | 7.85 | 26.41 |
| | 3 | 1.18 | 3.73 | 5.21 | 9.31 | 27.77 |
| ZHONG69 | 1 | 0.47 | 1.91 | 3.21 | 5.17 | 22.26 |
| | 2 | 0.26 | 3.08 | 3.64 | 5.61 | 21.07 |
| | 3 | 0.51 | 3.09 | 3.73 | 5.77 | 22.33 |

| | | | | | | |
|---|---|---|---|---|---|---|
| ZONG31 | 1 | 1.33 | 3.42 | 3.26 | 7.46 | 29.29 |
| | 2 | 0.73 | 3.57 | 3.39 | 7.16 | 29.09 |
| | 3 | 0.88 | 3.50 | 3.33 | 6.56 | 30.77 |
| ZZ01 | 1 | 1.82 | 1.41 | 3.83 | 6.20 | 26.20 |
| | 2 | 2.00 | 1.71 | 5.03 | 6.77 | 21.89 |
| | 3 | 1.88 | 1.86 | 4.88 | 6.11 | 24.72 |
| ZZ03 | 1 | 1.43 | 2.75 | 5.19 | 4.05 | 21.78 |
| | 2 | 1.66 | 3.48 | 5.05 | 5.41 | 20.58 |
| | 3 | 2.50 | 3.43 | 5.87 | 4.13 | 22.02 |
| 501 | 1 | 1.01 | 3.21 | 7.89 | 6.43 | 33.59 |
| | 2 | 0.82 | 4.09 | 8.07 | 6.02 | 32.36 |
| | 3 | 0.70 | 3.95 | 7.47 | 6.83 | 33.64 |
| 812 | 1 | 1.01 | 2.04 | 4.60 | 7.10 | 25.63 |
| | 2 | 0.60 | 5.35 | 6.19 | 6.61 | 22.37 |
| | 3 | 0.46 | 4.14 | 6.59 | 7.35 | 24.67 |
| 1323 | 1 | 0.16 | 2.91 | 2.82 | 3.51 | 30.27 |
| | 2 | 0.34 | 2.68 | 4.22 | 4.28 | 29.25 |
| | 3 | 0.25 | 2.79 | 3.52 | 4.27 | 30.43 |
| 3411 | 1 | 0.47 | 2.85 | 9.16 | 6.03 | 22.90 |
| | 2 | 0.60 | 4.09 | 8.86 | 5.81 | 23.14 |
| | 3 | 0.68 | 3.62 | 8.11 | 5.47 | 25.19 |
| 04K5672 | 1 | 0.52 | 2.54 | 4.67 | 4.44 | 33.67 |
| | 2 | 0.60 | 2.66 | 2.98 | 4.79 | 32.91 |
| | 3 | 0.56 | 3.05 | 3.22 | 4.01 | 33.96 |
| 04K5702 | 1 | 0.95 | 2.30 | 5.31 | 5.16 | 28.98 |
| | 2 | 1.09 | 2.52 | 2.86 | 5.06 | 27.04 |
| | 3 | 1.02 | 2.41 | 4.17 | 5.47 | 29.44 |
| 384-2 | 1 | 2.06 | 2.88 | 4.32 | 5.08 | 26.63 |
| | 2 | 1.14 | 3.05 | 4.15 | 5.53 | 24.36 |
| | 3 | 1.60 | 2.82 | 4.84 | 4.56 | 26.16 |
| BGY | 1 | 0.80 | 3.49 | 4.06 | 6.59 | 25.00 |
| | 2 | 0.43 | 6.08 | 4.21 | 5.61 | 25.68 |
| | 3 | 0.31 | 4.48 | 4.14 | 6.29 | 28.42 |
| BY843 | 1 | 0.44 | 2.12 | 5.83 | 6.25 | 32.91 |
| | 2 | 0.79 | 1.78 | 6.41 | 6.88 | 28.64 |
| | 3 | 0.92 | 1.82 | 5.82 | 6.07 | 32.36 |
| CF3 | 1 | 1.04 | 3.16 | 4.68 | 6.40 | 33.59 |
| | 2 | 0.75 | 2.67 | 2.87 | 6.82 | 30.61 |
| | 3 | 0.75 | 3.07 | 2.57 | 6.16 | 32.93 |
| CIMBL110 | 1 | 1.36 | 1.87 | 4.38 | 3.53 | 25.29 |
| | 2 | 0.90 | 2.06 | 4.26 | 3.41 | 25.21 |
| | 3 | 1.09 | 1.82 | 5.22 | 3.77 | 26.67 |
| CIMBL112 | 1 | 1.69 | 2.01 | 6.08 | 5.26 | 27.75 |
| | 2 | 1.35 | 2.15 | 6.47 | 4.17 | 21.54 |

| | | | | | | |
|---|---|---|---|---|---|---|
| | 3 | 1.22 | 2.08 | 6.72 | 4.00 | 25.48 |
| CIMBL117 | 1 | 0.95 | 2.11 | 4.07 | 2.27 | 30.92 |
| | 2 | 0.48 | 2.32 | 4.05 | 2.02 | 31.05 |
| | 3 | 0.41 | 2.21 | 4.06 | 2.82 | 32.48 |
| CIMBL134 | 1 | 1.46 | 1.03 | 6.11 | 5.17 | 15.15 |
| | 2 | 0.74 | 1.40 | 6.35 | 5.03 | 15.49 |
| | 3 | 0.80 | 1.81 | 6.53 | 5.78 | 15.99 |
| CIMBL136 | 1 | 1.68 | 2.09 | 2.98 | 6.44 | 28.16 |
| | 2 | 0.94 | 2.28 | 3.00 | 5.52 | 26.35 |
| | 3 | 1.16 | 2.64 | 2.69 | 6.35 | 27.91 |
| CIMBL138 | 1 | 1.07 | 3.33 | 2.91 | 6.24 | 23.03 |
| | 2 | 0.90 | 3.05 | 4.71 | 6.04 | 21.49 |
| | 3 | 0.54 | 3.19 | 3.81 | 6.37 | 21.59 |
| CIMBL146 | 1 | 1.68 | 2.93 | 5.33 | 6.59 | 22.41 |
| | 2 | 1.28 | 4.14 | 4.82 | 6.48 | 21.42 |
| | 3 | 1.18 | 5.03 | 5.52 | 5.79 | 21.25 |
| CIMBL154 | 1 | 1.40 | 2.44 | 2.75 | 5.52 | 28.60 |
| | 2 | 1.70 | 2.55 | 4.09 | 5.64 | 26.47 |
| | 3 | 1.70 | 2.50 | 3.72 | 6.33 | 28.95 |
| CIMBL30 | 1 | 0.67 | 1.77 | 5.21 | 4.97 | 28.74 |
| | 2 | 0.75 | 2.08 | 4.76 | 4.45 | 28.37 |
| | 3 | 0.86 | 2.52 | 6.04 | 4.56 | 29.39 |
| CIMBL34 | 1 | 2.01 | 2.72 | 5.06 | 6.92 | 30.28 |
| | 2 | 1.97 | 4.36 | 4.81 | 5.50 | 27.45 |
| | 3 | 0.95 | 4.14 | 4.33 | 5.61 | 30.11 |
| CIMBL45 | 1 | 0.15 | 2.37 | 9.12 | 4.88 | 40.31 |
| | 2 | 0.11 | 1.89 | 9.14 | 6.02 | 38.00 |
| | 3 | 0.17 | 1.68 | 9.13 | 5.68 | 40.65 |
| CIMBL57 | 1 | 1.04 | 1.99 | 7.12 | 4.60 | 23.50 |
| | 2 | 0.91 | 4.02 | 6.30 | 6.01 | 23.27 |
| | 3 | 0.97 | 4.50 | 7.01 | 5.83 | 25.69 |
| CIMBL67 | 1 | 0.79 | 2.03 | 7.11 | 5.10 | 27.31 |
| | 2 | 1.03 | 1.73 | 7.53 | 3.84 | 25.76 |
| | 3 | 0.76 | 2.03 | 7.02 | 4.25 | 27.37 |
| CIMBL8 | 1 | 0.67 | 5.21 | 2.76 | 6.56 | 26.15 |
| | 2 | 0.37 | 6.00 | 2.80 | 6.05 | 25.63 |
| | 3 | 0.22 | 5.76 | 2.78 | 7.06 | 26.72 |
| CIMBL80 | 1 | 0.62 | 1.61 | 4.51 | 4.54 | 22.11 |
| | 2 | 0.69 | 3.09 | 6.33 | 4.19 | 20.19 |
| | 3 | 0.65 | 2.65 | 5.87 | 4.59 | 21.98 |
| CIMBL85 | 1 | 0.89 | 4.37 | 5.67 | 5.90 | 42.33 |
| | 2 | 0.65 | 3.72 | 6.23 | 6.06 | 38.41 |
| | 3 | 0.66 | 4.34 | 5.80 | 5.49 | 41.12 |
| D047 | 1 | 0.80 | 3.33 | 3.93 | 5.39 | 35.98 |

| | | | | | | |
|---|---|---|---|---|---|---|
| | 2 | 1.11 | 3.63 | 3.66 | 5.11 | 32.79 |
| | 3 | 1.20 | 2.88 | 3.80 | 5.56 | 35.73 |
| DAN598 | 1 | 1.90 | 3.20 | 2.76 | 5.55 | 31.39 |
| | 2 | 0.76 | 4.47 | 3.59 | 5.61 | 26.74 |
| | 3 | 1.03 | 4.13 | 2.57 | 6.04 | 30.47 |
| DH29 | 1 | 1.08 | 0.83 | 5.98 | 6.59 | 15.90 |
| | 2 | 1.57 | 1.51 | 6.44 | 6.16 | 13.33 |
| | 3 | 1.78 | 0.87 | 6.75 | 7.12 | 13.86 |
| DSB | 1 | 2.13 | 5.86 | 5.41 | 3.80 | 19.60 |
| | 2 | 0.99 | 5.06 | 7.80 | 4.33 | 19.81 |
| | 3 | 2.02 | 5.46 | 5.86 | 4.82 | 20.53 |
| GEMS12 | 1 | 1.54 | 2.53 | 4.17 | 7.64 | 24.20 |
| | 2 | 2.04 | 2.30 | 4.21 | 8.13 | 23.49 |
| | 3 | 2.69 | 2.42 | 4.94 | 8.64 | 24.01 |
| GEMS27 | 1 | 0.85 | 2.29 | 4.02 | 3.54 | 27.75 |
| | 2 | 0.72 | 4.10 | 3.85 | 3.04 | 26.56 |
| | 3 | 0.78 | 3.95 | 4.68 | 3.87 | 27.82 |
| GEMS53 | 1 | 1.53 | 1.47 | 8.11 | 6.07 | 24.10 |
| | 2 | 2.33 | 4.07 | 8.15 | 6.14 | 23.00 |
| | 3 | 2.23 | 3.37 | 8.13 | 6.48 | 24.21 |
| GY220 | 1 | 1.07 | 3.07 | 3.37 | 6.21 | 29.11 |
| | 2 | 1.06 | 2.69 | 6.01 | 6.59 | 25.66 |
| | 3 | 1.06 | 3.18 | 5.86 | 6.78 | 28.97 |
| GY237 | 1 | 1.28 | 3.07 | 4.17 | 4.46 | 20.91 |
| | 2 | 0.65 | 3.52 | 3.58 | 5.04 | 19.52 |
| | 3 | 0.81 | 3.75 | 4.78 | 4.53 | 21.05 |
| GY246 | 1 | 2.23 | 2.15 | 3.70 | 7.01 | 24.48 |
| | 2 | 1.53 | 3.39 | 5.03 | 6.56 | 22.46 |
| | 3 | 1.43 | 3.22 | 4.07 | 7.17 | 24.22 |
| HB | 1 | 1.00 | 2.76 | 5.20 | 4.87 | 28.55 |
| | 2 | 0.30 | 3.34 | 4.64 | 5.87 | 26.09 |
| | 3 | 0.35 | 3.49 | 5.22 | 6.12 | 28.07 |
| HU803 | 1 | 1.38 | 3.29 | 4.23 | 7.45 | 21.37 |
| | 2 | 0.84 | 4.21 | 3.67 | 7.43 | 19.88 |
| | 3 | 0.51 | 4.18 | 3.85 | 7.72 | 21.29 |
| M165 | 1 | 1.06 | 2.83 | 7.70 | 7.69 | 26.74 |
| | 2 | 0.67 | 2.97 | 9.09 | 8.50 | 24.35 |
| | 3 | 1.02 | 2.45 | 9.00 | 8.55 | 26.38 |
| MN | 1 | 1.45 | 3.06 | 2.15 | 5.91 | 27.37 |
| | 2 | 1.34 | 3.03 | 4.05 | 7.92 | 23.39 |
| | 3 | 2.00 | 3.04 | 3.25 | 7.37 | 26.22 |
| QI319 | 1 | 1.20 | 1.71 | 4.34 | 10.45 | 28.99 |
| | 2 | 0.90 | 2.21 | 3.78 | 10.86 | 27.21 |
| | 3 | 0.91 | 2.11 | 4.21 | 11.11 | 29.51 |

| | | | | | | | |
|---|---|---|---|---|---|---|---|
| | R08 | 1 | 1.01 | 4.34 | 5.18 | 5.68 | 21.11 |
| | | 2 | 0.36 | 3.98 | 3.89 | 6.08 | 20.57 |
| | | 3 | 0.43 | 4.01 | 4.36 | 6.33 | 21.39 |
| | SHEN137 | 1 | 0.72 | 2.81 | 3.25 | 8.15 | 32.33 |
| | | 2 | 0.71 | 2.36 | 1.94 | 7.99 | 29.96 |
| | | 3 | 1.02 | 2.44 | 2.30 | 9.27 | 31.91 |
| | SI444 | 1 | 1.51 | 3.53 | 4.18 | 6.93 | 36.65 |
| | | 2 | 1.03 | 3.34 | 2.98 | 9.10 | 33.18 |
| | | 3 | 1.57 | 3.88 | 3.43 | 8.54 | 35.75 |
| | SY1077 | 1 | 1.70 | 3.62 | 8.33 | 11.44 | 26.18 |
| | | 2 | 1.54 | 3.66 | 6.96 | 10.51 | 23.46 |
| | | 3 | 1.24 | 3.64 | 7.05 | 10.90 | 25.57 |
| | SY999 | 1 | 1.40 | 2.26 | 6.12 | 8.74 | 23.83 |
| | | 2 | 1.37 | 2.44 | 6.22 | 9.03 | 22.89 |
| | | 3 | 0.94 | 2.65 | 7.07 | 9.34 | 24.28 |
| | TT16 | 1 | 1.34 | 2.54 | 3.46 | 5.14 | 28.64 |
| | | 2 | 1.12 | 2.89 | 4.06 | 5.68 | 26.68 |
| | | 3 | 0.91 | 2.41 | 3.16 | 5.93 | 28.07 |
| | TY10 | 1 | 1.57 | 0.46 | 6.04 | 4.75 | 29.55 |
| | | 2 | 2.23 | 2.06 | 4.99 | 5.54 | 27.38 |
| | | 3 | 1.54 | 2.61 | 5.05 | 5.89 | 29.07 |
| | TY7 | 1 | 2.05 | 2.64 | 5.53 | 4.69 | 22.29 |
| | | 2 | 1.56 | 3.06 | 7.00 | 5.92 | 22.10 |
| | | 3 | 1.97 | 3.00 | 5.82 | 5.01 | 23.45 |
| | TY8 | 1 | 1.62 | 0.93 | 3.98 | 8.54 | 33.98 |
| | | 2 | 2.75 | 2.09 | 5.12 | 8.44 | 40.01 |
| | | 3 | 2.49 | 1.96 | 4.97 | 9.24 | 31.16 |
| | WMR | 1 | 1.92 | 3.28 | 4.26 | 3.57 | 26.05 |
| | | 2 | 2.20 | 4.44 | 4.50 | 3.84 | 25.30 |
| | | 3 | 2.36 | 4.46 | 4.98 | 3.29 | 26.28 |
| | ZHENG58 | 1 | 0.12 | 2.77 | 6.01 | 4.73 | 31.56 |
| | | 2 | 0.25 | 3.29 | 7.06 | 4.68 | 31.73 |
| | | 3 | 0.19 | 3.78 | 7.29 | 4.68 | 33.22 |
| Changge | 177 | 1 | 0.67 | 1.97 | 4.77 | 6.87 | 28.79 |
| | | 2 | 0.80 | 3.16 | 3.38 | 7.87 | 25.92 |
| | | 3 | 0.87 | 2.51 | 4.40 | 6.81 | 25.68 |
| | 238 | 1 | 0.98 | 2.58 | 3.99 | 6.05 | 30.02 |
| | | 2 | 1.52 | 2.09 | 2.59 | 6.60 | 32.00 |
| | | 3 | 1.25 | 2.70 | 3.05 | 6.47 | 29.34 |
| | 268 | 1 | 1.27 | 2.14 | 4.57 | 7.04 | 26.84 |
| | | 2 | 1.59 | 2.69 | 4.66 | 7.02 | 26.14 |
| | | 3 | 1.04 | 2.31 | 4.21 | 7.36 | 25.76 |
| | 1462 | 1 | 1.08 | 2.28 | 3.83 | 6.20 | 25.57 |
| | | 2 | 0.96 | 2.43 | 4.29 | 7.20 | 25.34 |

| | | | | | | |
|---|---|---|---|---|---|---|
| | 3 | 1.53 | 2.67 | 4.74 | 5.31 | 25.75 |
| 4019 | 1 | 1.69 | 2.29 | 5.44 | 7.14 | 26.34 |
| | 2 | 2.01 | 1.94 | 4.30 | 6.82 | 23.61 |
| | 3 | 1.70 | 3.36 | 4.21 | 6.66 | 24.60 |
| 5213 | 1 | 0.62 | 4.47 | 4.30 | 5.96 | 27.17 |
| | 2 | 0.67 | 4.02 | 4.33 | 5.85 | 28.62 |
| | 3 | 1.97 | 4.81 | 4.28 | 6.82 | 28.55 |
| 9642 | 1 | 0.62 | 2.29 | 3.62 | 6.39 | 28.73 |
| | 2 | 0.64 | 2.52 | 3.79 | 5.44 | 25.21 |
| | 3 | 0.40 | 2.80 | 4.74 | 6.29 | 25.37 |
| 526018 | 1 | 0.98 | 1.90 | 3.98 | 5.89 | 26.50 |
| | 2 | 1.55 | 2.40 | 3.04 | 6.13 | 26.77 |
| | 3 | 1.19 | 2.35 | 3.14 | 5.88 | 25.62 |
| 05WN230 | 1 | 1.38 | 1.71 | 4.90 | 5.53 | 25.08 |
| | 2 | 0.87 | 1.99 | 5.34 | 5.83 | 26.45 |
| | 3 | 0.92 | 2.53 | 5.64 | 6.55 | 30.36 |
| 4F1 | 1 | 0.97 | 3.13 | 4.15 | 6.85 | 27.62 |
| | 2 | 0.74 | 3.52 | 4.97 | 6.36 | 27.57 |
| | 3 | 0.87 | 3.98 | 5.29 | 5.81 | 26.27 |
| 7884-4HT | 1 | 1.46 | 2.89 | 4.19 | 6.45 | 28.51 |
| | 2 | 1.12 | 3.71 | 4.30 | 7.46 | 27.85 |
| | 3 | 1.24 | 3.96 | 5.48 | 7.29 | 24.07 |
| 835B | 1 | 0.98 | 3.17 | 3.75 | 5.69 | 24.52 |
| | 2 | 0.66 | 3.42 | 5.87 | 5.47 | 25.43 |
| | 3 | 2.86 | 2.91 | 5.05 | 5.95 | 28.91 |
| 975-12 | 1 | 2.06 | 2.61 | 6.23 | 7.69 | 23.08 |
| | 2 | 1.76 | 2.79 | 5.83 | 7.07 | 22.22 |
| | 3 | 2.06 | 2.47 | 6.25 | 7.01 | 22.28 |
| B110 | 1 | 0.74 | 3.42 | 6.04 | 7.28 | 34.20 |
| | 2 | 0.77 | 4.18 | 6.61 | 6.79 | 30.69 |
| | 3 | 1.15 | 3.36 | 7.21 | 6.61 | 35.26 |
| B111 | 1 | 1.13 | 3.30 | 4.95 | 5.94 | 31.62 |
| | 2 | 0.71 | 3.56 | 5.01 | 6.70 | 28.75 |
| | 3 | 1.21 | 2.80 | 4.95 | 6.47 | 33.04 |
| B151 | 1 | 1.89 | 3.37 | 4.19 | 6.44 | 27.19 |
| | 2 | 1.30 | 4.00 | 3.75 | 7.31 | 27.17 |
| | 3 | 1.71 | 3.49 | 3.72 | 6.37 | 27.68 |
| BS16 | 1 | 1.69 | 2.20 | 5.37 | 5.68 | 29.59 |
| | 2 | 1.83 | 1.93 | 6.04 | 5.74 | 28.72 |
| | 3 | 1.83 | 1.58 | 5.33 | 5.12 | 26.80 |
| BY4839 | 1 | 1.01 | 3.18 | 4.38 | 6.47 | 37.71 |
| | 2 | 0.70 | 3.53 | 4.10 | 7.17 | 43.62 |
| | 3 | 0.81 | 3.03 | 6.19 | 7.75 | 35.57 |
| BY804 | 1 | 1.32 | 2.44 | 2.77 | 6.49 | 29.03 |

| | | | | | | |
|---|---|---|---|---|---|---|
| | 2 | 0.85 | 2.71 | 2.21 | 5.64 | 27.58 |
| | 3 | 1.61 | 2.59 | 3.69 | 5.29 | 28.98 |
| BY807 | 1 | 0.63 | 1.45 | 4.38 | 3.33 | 22.96 |
| | 2 | 0.51 | 2.85 | 5.75 | 6.40 | 24.00 |
| | 3 | 0.62 | 2.49 | 4.81 | 6.22 | 28.30 |
| BY809 | 1 | 1.11 | 3.66 | 2.95 | 5.96 | 27.41 |
| | 2 | 1.06 | 4.20 | 2.38 | 6.74 | 26.02 |
| | 3 | 0.78 | 3.81 | 2.71 | 5.52 | 28.36 |
| BY813 | 1 | 1.11 | 2.37 | 5.09 | 6.38 | 33.26 |
| | 2 | 0.90 | 3.41 | 5.11 | 6.66 | 30.58 |
| | 3 | 1.15 | 2.80 | 4.07 | 6.89 | 32.01 |
| BY815 | 1 | 2.67 | 3.66 | 5.11 | 7.53 | 27.13 |
| | 2 | 1.95 | 3.06 | 6.16 | 8.29 | 25.64 |
| | 3 | 2.00 | 2.97 | 4.61 | 8.16 | 26.14 |
| BY855 | 1 | 1.10 | 2.98 | 4.97 | 5.96 | 26.00 |
| | 2 | 0.61 | 2.95 | 4.05 | 5.05 | 28.41 |
| | 3 | 0.79 | 3.31 | 5.57 | 7.31 | 22.48 |
| CHUAN48-2 | 1 | 1.65 | 3.61 | 4.34 | 5.92 | 23.41 |
| | 2 | 1.48 | 3.29 | 4.27 | 6.53 | 23.50 |
| | 3 | 1.64 | 3.60 | 4.31 | 5.85 | 23.45 |
| CIMBL1 | 1 | 2.06 | 2.09 | 3.83 | 7.48 | 23.52 |
| | 2 | 1.13 | 2.27 | 3.27 | 8.08 | 26.21 |
| | 3 | 1.25 | 2.62 | 3.90 | 5.96 | 26.00 |
| CIMBL105 | 1 | 0.86 | 3.79 | 3.07 | 5.42 | 29.10 |
| | 2 | 0.84 | 3.60 | 3.23 | 5.41 | 24.71 |
| | 3 | 0.85 | 3.11 | 3.65 | 5.70 | 26.16 |
| CIMBL106 | 1 | 1.11 | 1.78 | 4.72 | 4.43 | 33.90 |
| | 2 | 0.80 | 1.85 | 4.00 | 5.29 | 31.37 |
| | 3 | 0.91 | 2.11 | 4.79 | 5.36 | 30.71 |
| CIMBL109 | 1 | 1.09 | 2.46 | 5.39 | 6.88 | 30.75 |
| | 2 | 1.06 | 2.66 | 4.48 | 6.73 | 28.85 |
| | 3 | 0.47 | 3.33 | 4.60 | 7.30 | 29.96 |
| CIMBL11 | 1 | 1.61 | 2.34 | 4.07 | 5.30 | 19.27 |
| | 2 | 1.89 | 2.97 | 5.96 | 5.15 | 14.26 |
| | 3 | 1.18 | 2.53 | 4.63 | 5.58 | 24.08 |
| CIMBL12 | 1 | 1.31 | 2.93 | 3.84 | 6.79 | 32.19 |
| | 2 | 0.71 | 2.56 | 3.08 | 7.02 | 26.17 |
| | 3 | 1.33 | 3.22 | 4.60 | 7.39 | 27.89 |
| CIMBL120 | 1 | 1.53 | 3.27 | 4.84 | 5.40 | 28.52 |
| | 2 | 1.10 | 4.77 | 4.92 | 5.82 | 28.52 |
| | 3 | 0.92 | 3.90 | 4.20 | 5.91 | 28.52 |
| CIMBL123 | 1 | 1.06 | 3.01 | 4.16 | 5.51 | 25.98 |
| | 2 | 1.37 | 2.69 | 4.56 | 6.89 | 25.94 |
| | 3 | 0.74 | 3.38 | 3.69 | 5.80 | 22.64 |

| | | | | | | |
|---|---|---|---|---|---|---|
| CIMBL124 | 1 | 1.29 | 2.79 | 4.26 | 5.44 | 28.10 |
| | 2 | 0.92 | 2.67 | 5.59 | 5.80 | 26.58 |
| | 3 | 1.18 | 2.99 | 4.64 | 5.09 | 28.29 |
| CIMBL127 | 1 | 0.67 | 2.07 | 4.51 | 5.37 | 31.08 |
| | 2 | 0.38 | 2.39 | 3.40 | 5.32 | 33.37 |
| | 3 | 1.13 | 2.52 | 4.75 | 6.67 | 31.58 |
| CIMBL133 | 1 | 1.12 | 2.48 | 3.34 | 5.93 | 18.16 |
| | 2 | 1.17 | 2.34 | 2.46 | 6.03 | 19.31 |
| | 3 | 0.75 | 2.90 | 2.48 | 6.31 | 23.59 |
| CIMBL140 | 1 | 1.65 | 4.12 | 4.23 | 7.01 | 32.52 |
| | 2 | 1.86 | 4.13 | 5.49 | 7.58 | 28.98 |
| | 3 | 1.82 | 4.44 | 5.81 | 9.26 | 28.23 |
| CIMBL142 | 1 | 0.89 | 2.54 | 3.67 | 6.38 | 29.91 |
| | 2 | 0.73 | 2.95 | 2.68 | 6.21 | 28.55 |
| | 3 | 0.62 | 3.07 | 2.57 | 6.67 | 30.40 |
| CIMBL143 | 1 | 2.24 | 3.01 | 3.94 | 5.81 | 28.19 |
| | 2 | 1.78 | 3.47 | 5.15 | 4.40 | 27.50 |
| | 3 | 2.43 | 2.78 | 3.54 | 5.65 | 26.31 |
| CIMBL144 | 1 | 0.43 | 3.02 | 3.66 | 7.07 | 23.98 |
| | 2 | 0.89 | 2.68 | 3.14 | 6.79 | 27.15 |
| | 3 | 0.77 | 2.78 | 3.31 | 6.56 | 26.32 |
| CIMBL150 | 1 | 1.55 | 3.16 | 3.60 | 9.42 | 29.90 |
| | 2 | 2.03 | 2.84 | 3.67 | 7.72 | 27.00 |
| | 3 | 1.52 | 2.89 | 3.86 | 5.19 | 29.56 |
| CIMBL151 | 1 | 1.15 | 2.79 | 3.62 | 6.10 | 25.25 |
| | 2 | 1.09 | 4.99 | 6.44 | 6.92 | 25.66 |
| | 3 | 0.96 | 3.46 | 4.04 | 7.14 | 25.80 |
| CIMBL153 | 1 | 0.55 | 2.32 | 4.16 | 5.83 | 28.82 |
| | 2 | 0.53 | 2.43 | 2.85 | 5.35 | 28.97 |
| | 3 | 1.20 | 2.87 | 4.18 | 5.32 | 31.07 |
| CIMBL157 | 1 | 1.20 | 3.15 | 2.47 | 6.70 | 22.81 |
| | 2 | 0.95 | 3.96 | 4.05 | 7.06 | 23.96 |
| | 3 | 0.90 | 3.40 | 3.44 | 6.50 | 23.76 |
| CIMBL2 | 1 | 1.74 | 2.08 | 5.53 | 8.57 | 26.31 |
| | 2 | 0.93 | 2.84 | 7.37 | 8.75 | 27.63 |
| | 3 | 1.11 | 2.08 | 6.70 | 8.52 | 29.72 |
| CIMBL21 | 1 | 1.01 | 2.14 | 3.15 | 5.70 | 34.67 |
| | 2 | 0.79 | 2.43 | 2.74 | 5.96 | 32.76 |
| | 3 | 1.31 | 2.55 | 3.49 | 5.55 | 34.26 |
| CIMBL23 | 1 | 1.10 | 2.32 | 3.97 | 5.26 | 24.44 |
| | 2 | 0.69 | 2.74 | 3.80 | 5.62 | 23.18 |
| | 3 | 0.89 | 2.61 | 4.18 | 5.14 | 24.18 |
| CIMBL32 | 1 | 1.19 | 2.20 | 3.30 | 4.87 | 26.42 |
| | 2 | 1.62 | 2.61 | 6.09 | 4.19 | 27.76 |

| | | | | | | |
|---|---|---|---|---|---|---|
| | 3 | 0.97 | 2.81 | 2.83 | 4.34 | 27.63 |
| CIMBL38 | 1 | 0.70 | 2.27 | 4.67 | 6.85 | 28.74 |
| | 2 | 0.92 | 2.00 | 4.13 | 7.55 | 28.12 |
| | 3 | 1.19 | 2.16 | 4.67 | 5.40 | 30.83 |
| CIMBL42 | 1 | 0.52 | 2.65 | 6.09 | 7.80 | 27.62 |
| | 2 | 0.86 | 2.60 | 7.56 | 8.05 | 27.29 |
| | 3 | 0.45 | 2.61 | 6.97 | 7.06 | 27.50 |
| CIMBL48 | 1 | 1.27 | 2.76 | 5.20 | 6.73 | 31.02 |
| | 2 | 1.12 | 3.30 | 5.76 | 5.27 | 31.24 |
| | 3 | 0.71 | 2.86 | 4.79 | 5.84 | 34.47 |
| CIMBL49 | 1 | 0.97 | 2.32 | 4.88 | 6.23 | 32.89 |
| | 2 | 0.78 | 2.50 | 3.37 | 6.13 | 29.83 |
| | 3 | 0.88 | 2.67 | 2.79 | 6.28 | 28.58 |
| CIMBL58 | 1 | 0.77 | 2.87 | 4.06 | 4.14 | 31.68 |
| | 2 | 0.83 | 3.25 | 2.33 | 4.42 | 33.15 |
| | 3 | 0.72 | 3.29 | 3.56 | 4.57 | 29.00 |
| CIMBL59 | 1 | 1.71 | 2.39 | 3.33 | 5.30 | 30.71 |
| | 2 | 1.27 | 2.64 | 4.30 | 5.29 | 30.88 |
| | 3 | 1.26 | 2.59 | 3.97 | 5.88 | 40.47 |
| CIMBL60 | 1 | 1.04 | 2.99 | 3.95 | 5.35 | 22.07 |
| | 2 | 1.01 | 3.34 | 4.00 | 4.70 | 23.17 |
| | 3 | 0.94 | 2.78 | 5.55 | 4.91 | 23.55 |
| CIMBL75 | 1 | 1.28 | 2.89 | 2.93 | 6.01 | 31.30 |
| | 2 | 1.74 | 2.57 | 5.01 | 5.13 | 29.59 |
| | 3 | 1.91 | 3.10 | 3.19 | 6.89 | 30.99 |
| CIMBL77 | 1 | 0.62 | 2.95 | 4.07 | 5.12 | 27.58 |
| | 2 | 0.45 | 3.24 | 2.43 | 5.17 | 26.77 |
| | 3 | 0.84 | 3.15 | 3.38 | 5.02 | 27.48 |
| CIMBL84 | 1 | 1.04 | 2.11 | 3.59 | 5.85 | 25.33 |
| | 2 | 0.80 | 2.22 | 3.68 | 5.68 | 21.97 |
| | 3 | 0.54 | 2.80 | 4.32 | 5.20 | 25.30 |
| CIMBL86 | 1 | 0.96 | 1.89 | 4.44 | 5.04 | 23.07 |
| | 2 | 0.82 | 2.93 | 5.36 | 5.48 | 24.96 |
| | 3 | 1.20 | 2.84 | 4.78 | 6.03 | 26.14 |
| CIMBL87 | 1 | 1.77 | 2.61 | 3.25 | 5.78 | 26.43 |
| | 2 | 1.89 | 2.41 | 7.34 | 5.96 | 22.83 |
| | 3 | 1.24 | 2.24 | 3.69 | 6.33 | 27.96 |
| CIMBL88 | 1 | 1.28 | 2.08 | 3.12 | 6.77 | 25.03 |
| | 2 | 1.66 | 2.80 | 1.42 | 6.59 | 26.57 |
| | 3 | 1.64 | 3.16 | 3.04 | 7.23 | 28.79 |
| CIMBL91 | 1 | 1.07 | 3.01 | 5.40 | 7.15 | 28.47 |
| | 2 | 1.79 | 3.23 | 4.96 | 7.29 | 30.15 |
| | 3 | 1.54 | 3.02 | 4.66 | 7.47 | 28.18 |
| CIMBL92 | 1 | 0.88 | 2.71 | 3.27 | 5.13 | 27.21 |

| | | | | | | |
|---|---|---|---|---|---|---|
| | 2 | 0.84 | 2.74 | 2.68 | 5.56 | 27.37 |
| | 3 | 0.85 | 2.42 | 3.08 | 5.50 | 29.52 |
| CIMBL93 | 1 | 1.70 | 2.91 | 4.07 | 5.65 | 31.58 |
| | 2 | 1.55 | 3.78 | 3.03 | 6.04 | 29.60 |
| | 3 | 1.50 | 3.44 | 4.61 | 5.28 | 30.03 |
| CIMBL96 | 1 | 1.12 | 3.65 | 4.50 | 6.31 | 26.25 |
| | 2 | 1.40 | 3.73 | 3.14 | 6.95 | 25.85 |
| | 3 | 1.00 | 3.36 | 4.80 | 6.16 | 26.42 |
| CML115 | 1 | 1.61 | 3.08 | 4.94 | 6.21 | 30.62 |
| | 2 | 1.52 | 1.90 | 6.43 | 7.49 | 31.78 |
| | 3 | 2.47 | 2.20 | 4.11 | 6.82 | 30.01 |
| CML116 | 1 | 0.97 | 1.48 | 3.44 | 4.68 | 30.31 |
| | 2 | 0.70 | 2.16 | 2.91 | 4.70 | 27.42 |
| | 3 | 0.95 | 1.74 | 2.55 | 5.17 | 34.28 |
| CML118 | 1 | 0.86 | 3.60 | 3.94 | 6.47 | 27.52 |
| | 2 | 1.14 | 3.69 | 3.91 | 6.16 | 29.74 |
| | 3 | 0.84 | 3.90 | 4.86 | 6.76 | 31.07 |
| CML121 | 1 | 0.74 | 2.43 | 4.65 | 4.68 | 25.29 |
| | 2 | 0.56 | 2.56 | 4.81 | 5.35 | 24.50 |
| | 3 | 0.48 | 2.27 | 4.72 | 5.51 | 24.09 |
| CML122 | 1 | 1.30 | 3.96 | 5.63 | 5.65 | 31.13 |
| | 2 | 0.74 | 2.18 | 5.08 | 5.04 | 26.17 |
| | 3 | 2.00 | 2.40 | 4.80 | 5.42 | 30.25 |
| CML130 | 1 | 0.82 | 2.75 | 7.02 | 6.68 | 27.37 |
| | 2 | 0.60 | 2.74 | 7.17 | 6.36 | 24.80 |
| | 3 | 0.96 | 3.04 | 6.40 | 6.25 | 32.97 |
| CML139 | 1 | 1.36 | 2.31 | 4.32 | 4.83 | 28.52 |
| | 2 | 1.67 | 3.31 | 3.31 | 4.93 | 25.40 |
| | 3 | 1.22 | 2.11 | 4.97 | 5.40 | 25.12 |
| CML163 | 1 | 1.90 | 2.97 | 3.48 | 6.58 | 22.90 |
| | 2 | 1.76 | 5.48 | 2.53 | 6.13 | 26.01 |
| | 3 | 1.12 | 3.55 | 2.84 | 5.58 | 23.29 |
| CML165 | 1 | 0.94 | 3.21 | 3.88 | 4.92 | 25.21 |
| | 2 | 0.65 | 4.68 | 4.22 | 5.34 | 28.22 |
| | 3 | 0.80 | 3.67 | 4.63 | 3.21 | 26.79 |
| CML170 | 1 | 0.79 | 2.32 | 3.40 | 5.16 | 32.18 |
| | 2 | 0.73 | 2.21 | 2.64 | 5.91 | 32.73 |
| | 3 | 1.04 | 1.60 | 3.61 | 5.92 | 28.76 |
| CML290 | 1 | 0.89 | 3.27 | 3.34 | 6.71 | 27.13 |
| | 2 | 0.49 | 3.55 | 4.02 | 7.48 | 27.03 |
| | 3 | 0.85 | 3.49 | 4.05 | 6.32 | 26.19 |
| CML304 | 1 | 1.17 | 2.77 | 4.82 | 5.40 | 29.41 |
| | 2 | 1.84 | 2.97 | 2.10 | 4.76 | 26.79 |
| | 3 | 0.80 | 2.91 | 4.28 | 4.73 | 29.64 |

| | | | | | | |
|---|---|---|---|---|---|---|
| CML31 | 1 | 0.65 | 2.87 | 4.57 | 5.42 | 25.37 |
| | 2 | 0.54 | 2.63 | 4.90 | 5.65 | 24.82 |
| | 3 | 0.82 | 2.65 | 4.61 | 5.82 | 25.60 |
| CML325 | 1 | 1.29 | 3.07 | 4.02 | 5.41 | 32.25 |
| | 2 | 0.57 | 3.63 | 5.52 | 6.33 | 29.14 |
| | 3 | 0.86 | 3.68 | 4.02 | 5.83 | 28.05 |
| CML338 | 1 | 4.13 | 2.96 | 5.77 | 4.66 | 23.96 |
| | 2 | 3.42 | 4.16 | 6.66 | 4.86 | 23.50 |
| | 3 | 4.30 | 3.51 | 3.98 | 5.48 | 24.02 |
| CML423 | 1 | 0.70 | 2.76 | 6.21 | 5.98 | 30.98 |
| | 2 | 1.10 | 3.28 | 3.42 | 5.78 | 32.29 |
| | 3 | 1.05 | 3.13 | 4.82 | 5.88 | 33.06 |
| CML479 | 1 | 0.95 | 2.55 | 4.19 | 4.50 | 26.91 |
| | 2 | 0.94 | 2.70 | 5.58 | 4.25 | 23.26 |
| | 3 | 0.62 | 3.28 | 4.00 | 4.55 | 29.04 |
| D863F | 1 | 0.58 | 2.96 | 5.90 | 6.50 | 23.01 |
| | 2 | 0.17 | 4.09 | 6.02 | 6.38 | 25.91 |
| | 3 | 0.30 | 3.39 | 4.68 | 6.49 | 28.55 |
| DAN3130 | 1 | 1.25 | 2.56 | 3.63 | 5.56 | 25.28 |
| | 2 | 1.56 | 2.80 | 4.04 | 5.45 | 29.92 |
| | 3 | 1.56 | 2.75 | 4.34 | 5.66 | 28.35 |
| DAN360 | 1 | 1.87 | 2.07 | 4.19 | 11.65 | 24.76 |
| | 2 | 1.15 | 2.42 | 7.84 | 9.89 | 27.22 |
| | 3 | 1.68 | 1.67 | 4.84 | 8.22 | 26.82 |
| DAN4245 | 1 | 0.87 | 3.08 | 5.48 | 7.88 | 25.60 |
| | 2 | 1.15 | 2.72 | 4.03 | 8.48 | 24.15 |
| | 3 | 0.96 | 3.60 | 4.46 | 8.73 | 25.15 |
| DAN599 | 1 | 0.55 | 2.57 | 3.92 | 5.63 | 22.08 |
| | 2 | 0.78 | 2.15 | 2.46 | 5.40 | 18.51 |
| | 3 | 0.46 | 2.77 | 4.31 | 5.83 | 27.93 |
| DH3732 | 1 | 1.23 | 4.44 | 3.55 | 6.64 | 24.90 |
| | 2 | 1.68 | 4.99 | 3.53 | 5.68 | 25.87 |
| | 3 | 0.91 | 3.80 | 3.11 | 5.75 | 31.39 |
| DONG46 | 1 | 1.82 | 2.38 | 4.70 | 5.46 | 34.76 |
| | 2 | 2.10 | 2.18 | 5.43 | 5.30 | 33.53 |
| | 3 | 1.25 | 2.97 | 4.35 | 5.32 | 34.16 |
| EN25 | 1 | 1.62 | 2.58 | 3.96 | 5.90 | 28.61 |
| | 2 | 1.83 | 3.33 | 4.63 | 5.56 | 27.44 |
| | 3 | 0.84 | 3.55 | 4.42 | 5.94 | 28.69 |
| ES40 | 1 | 1.29 | 2.79 | 3.12 | 5.11 | 26.50 |
| | 2 | 0.75 | 2.35 | 4.75 | 4.69 | 26.05 |
| | 3 | 0.93 | 2.87 | 3.05 | 4.86 | 26.00 |
| FCD0602 | 1 | 1.23 | 1.95 | 3.76 | 4.89 | 25.31 |
| | 2 | 1.70 | 2.81 | 4.32 | 6.07 | 22.94 |

| | | | | | | |
|---|---|---|---|---|---|---|
| | 3 | 1.44 | 2.19 | 3.51 | 6.79 | 25.25 |
| GEMS10 | 1 | 0.73 | 1.72 | 3.86 | 11.24 | 32.45 |
| | 2 | 0.76 | 2.26 | 5.98 | 9.91 | 30.26 |
| | 3 | 0.72 | 2.40 | 3.67 | 9.87 | 27.98 |
| GEMS11 | 1 | 1.09 | 3.90 | 3.79 | 7.62 | 26.10 |
| | 2 | 0.44 | 3.89 | 3.96 | 6.43 | 26.23 |
| | 3 | 1.77 | 4.03 | 4.55 | 6.22 | 35.86 |
| GEMS13 | 1 | 0.86 | 2.17 | 3.85 | 5.17 | 28.92 |
| | 2 | 0.66 | 2.71 | 3.84 | 5.26 | 28.77 |
| | 3 | 0.76 | 2.29 | 3.85 | 4.84 | 28.47 |
| GEMS14 | 1 | 1.10 | 2.81 | 2.99 | 5.55 | 30.57 |
| | 2 | 1.70 | 3.50 | 4.41 | 6.24 | 29.30 |
| | 3 | 1.21 | 2.19 | 3.20 | 6.07 | 29.90 |
| GEMS15 | 1 | 0.98 | 2.06 | 2.85 | 6.17 | 24.05 |
| | 2 | 1.21 | 1.88 | 6.86 | 6.94 | 23.04 |
| | 3 | 0.73 | 2.03 | 3.62 | 6.69 | 27.42 |
| GEMS17 | 1 | 2.17 | 2.86 | 3.48 | 4.90 | 25.17 |
| | 2 | 1.21 | 3.67 | 4.38 | 5.18 | 26.87 |
| | 3 | 1.84 | 3.79 | 3.62 | 5.26 | 26.39 |
| GEMS19 | 1 | 0.88 | 3.02 | 4.09 | 6.07 | 29.09 |
| | 2 | 0.81 | 2.84 | 4.20 | 5.83 | 26.71 |
| | 3 | 0.66 | 2.59 | 4.89 | 6.00 | 26.21 |
| GEMS21 | 1 | 0.50 | 3.74 | 4.15 | 5.71 | 29.19 |
| | 2 | 0.80 | 3.88 | 3.64 | 6.46 | 28.37 |
| | 3 | 0.73 | 3.40 | 3.51 | 7.07 | 32.45 |
| GEMS25 | 1 | 1.16 | 2.68 | 3.17 | 5.45 | 34.22 |
| | 2 | 1.29 | 2.71 | 7.42 | 6.14 | 26.64 |
| | 3 | 1.24 | 2.96 | 3.84 | 5.92 | 32.92 |
| GEMS28 | 1 | 2.30 | 3.22 | 5.97 | 4.96 | 26.49 |
| | 2 | 1.73 | 4.09 | 5.50 | 5.55 | 26.89 |
| | 3 | 1.80 | 3.99 | 5.92 | 5.97 | 30.70 |
| GEMS29 | 1 | 1.43 | 2.18 | 3.43 | 5.62 | 35.30 |
| | 2 | 1.38 | 2.43 | 3.06 | 5.62 | 33.06 |
| | 3 | 1.32 | 2.26 | 3.12 | 5.85 | 31.64 |
| GEMS3 | 1 | 0.86 | 2.21 | 4.43 | 5.49 | 26.64 |
| | 2 | 1.21 | 2.75 | 3.22 | 7.30 | 28.25 |
| | 3 | 1.91 | 3.27 | 5.10 | 8.00 | 25.50 |
| GEMS30 | 1 | 1.74 | 2.57 | 4.86 | 6.17 | 26.41 |
| | 2 | 1.43 | 3.81 | 2.87 | 5.35 | 30.95 |
| | 3 | 1.88 | 2.32 | 3.72 | 5.41 | 26.54 |
| GEMS31 | 1 | 1.22 | 2.26 | 4.19 | 4.81 | 30.75 |
| | 2 | 0.81 | 2.06 | 5.06 | 5.79 | 28.72 |
| | 3 | 2.39 | 2.27 | 3.69 | 4.90 | 31.99 |
| GEMS32 | 1 | 2.08 | 3.22 | 4.43 | 6.63 | 27.79 |

| | | | | | | |
|---|---|---|---|---|---|---|
| | 2 | 1.63 | 2.92 | 6.47 | 6.15 | 26.16 |
| | 3 | 1.24 | 2.91 | 4.81 | 7.45 | 26.57 |
| GEMS36 | 1 | 1.42 | 3.10 | 9.97 | 7.07 | 30.93 |
| | 2 | 1.03 | 2.41 | 7.22 | 7.26 | 25.32 |
| | 3 | 1.50 | 3.01 | 8.42 | 6.45 | 28.20 |
| GEMS39 | 1 | 1.11 | 2.44 | 5.17 | 6.89 | 30.04 |
| | 2 | 1.44 | 3.03 | 6.11 | 7.93 | 31.79 |
| | 3 | 0.86 | 3.56 | 2.94 | 5.47 | 29.58 |
| GEMS42 | 1 | 0.38 | 2.83 | 3.84 | 5.09 | 34.91 |
| | 2 | 0.50 | 3.99 | 5.46 | 10.10 | 27.54 |
| | 3 | 2.64 | 3.05 | 4.28 | 5.31 | 29.14 |
| GEMS44 | 1 | 1.12 | 3.33 | 4.49 | 7.44 | 28.51 |
| | 2 | 0.58 | 3.86 | 7.34 | 8.09 | 26.52 |
| | 3 | 0.75 | 4.11 | 5.10 | 7.84 | 32.96 |
| GEMS48 | 1 | 1.18 | 3.06 | 3.87 | 6.66 | 24.58 |
| | 2 | 1.97 | 2.32 | 5.56 | 5.94 | 26.13 |
| | 3 | 1.19 | 2.55 | 4.43 | 7.17 | 24.30 |
| GEMS49 | 1 | 1.14 | 3.10 | 3.24 | 6.67 | 33.54 |
| | 2 | 1.08 | 4.67 | 3.74 | 7.33 | 26.91 |
| | 3 | 1.57 | 3.76 | 3.64 | 6.84 | 23.48 |
| GEMS5 | 1 | 1.20 | 2.87 | 4.96 | 6.87 | 25.86 |
| | 2 | 1.17 | 2.36 | 4.12 | 6.81 | 23.49 |
| | 3 | 1.12 | 2.21 | 4.07 | 7.23 | 26.93 |
| GEMS51 | 1 | 1.09 | 2.75 | 6.74 | 6.23 | 24.89 |
| | 2 | 1.07 | 2.79 | 6.07 | 5.70 | 28.22 |
| | 3 | 1.15 | 3.02 | 6.16 | 6.96 | 28.21 |
| GEMS55 | 1 | 2.12 | 3.71 | 4.90 | 6.32 | 29.26 |
| | 2 | 1.61 | 3.49 | 5.35 | 5.86 | 27.20 |
| | 3 | 1.66 | 3.76 | 3.21 | 6.61 | 26.03 |
| GEMS58 | 1 | 0.62 | 2.60 | 4.22 | 5.22 | 27.34 |
| | 2 | 0.60 | 2.69 | 4.42 | 5.85 | 26.40 |
| | 3 | 0.79 | 2.48 | 4.71 | 4.36 | 27.79 |
| GEMS6 | 1 | 0.83 | 2.87 | 4.87 | 6.99 | 22.19 |
| | 2 | 0.91 | 3.74 | 5.73 | 6.78 | 23.63 |
| | 3 | 0.68 | 3.79 | 5.23 | 6.80 | 24.60 |
| GEMS61 | 1 | 1.87 | 2.74 | 3.05 | 5.51 | 26.13 |
| | 2 | 1.98 | 2.78 | 2.61 | 7.03 | 31.79 |
| | 3 | 2.21 | 3.53 | 2.55 | 5.92 | 24.98 |
| GEMS63 | 1 | 1.20 | 2.37 | 5.00 | 6.15 | 25.95 |
| | 2 | 0.84 | 2.86 | 6.04 | 6.65 | 22.17 |
| | 3 | 1.09 | 3.06 | 4.19 | 5.64 | 27.05 |
| GY462 | 1 | 1.16 | 3.22 | 3.63 | 6.87 | 30.29 |
| | 2 | 0.85 | 2.62 | 4.16 | 5.36 | 23.14 |
| | 3 | 1.07 | 3.07 | 5.26 | 5.74 | 27.09 |

| | | | | | | |
|---|---|---|---|---|---|---|
| GY923 | 1 | 0.67 | 2.07 | 2.67 | 6.69 | 29.92 |
| | 2 | 0.70 | 3.17 | 6.67 | 6.32 | 32.97 |
| | 3 | 0.24 | 3.25 | 3.24 | 6.26 | 30.12 |
| IRF314 | 1 | 0.96 | 3.32 | 4.67 | 7.80 | 28.98 |
| | 2 | 1.35 | 3.65 | 3.70 | 8.86 | 29.68 |
| | 3 | 1.48 | 4.12 | 3.65 | 7.59 | 30.05 |
| J4112 | 1 | 0.86 | 2.37 | 2.34 | 5.72 | 28.62 |
| | 2 | 0.54 | 2.64 | 3.58 | 5.66 | 26.02 |
| | 3 | 0.93 | 2.71 | 3.73 | 6.98 | 22.33 |
| JH59 | 1 | 0.68 | 3.47 | 3.86 | 5.29 | 31.23 |
| | 2 | 1.19 | 2.65 | 3.76 | 6.24 | 32.11 |
| | 3 | 0.75 | 3.17 | 3.51 | 5.38 | 31.35 |
| JH96C | 1 | 0.70 | 2.61 | 5.38 | 6.16 | 26.48 |
| | 2 | 0.81 | 2.66 | 5.17 | 6.63 | 26.72 |
| | 3 | 0.68 | 2.59 | 5.49 | 6.73 | 27.30 |
| JI846 | 1 | 2.12 | 2.80 | 1.69 | 5.70 | 25.15 |
| | 2 | 1.74 | 2.83 | 2.32 | 5.32 | 33.21 |
| | 3 | 2.20 | 3.71 | 2.98 | 5.76 | 28.63 |
| JI853 | 1 | 1.09 | 2.68 | 4.23 | 5.75 | 26.46 |
| | 2 | 0.85 | 2.63 | 4.51 | 6.69 | 31.79 |
| | 3 | 0.90 | 2.32 | 4.45 | 8.10 | 26.47 |
| JY01 | 1 | 0.74 | 2.48 | 3.32 | 7.21 | 25.79 |
| | 2 | 0.72 | 2.48 | 3.31 | 7.33 | 24.04 |
| | 3 | 0.95 | 2.75 | 4.41 | 7.45 | 27.38 |
| K10 | 1 | 1.92 | 2.74 | 4.85 | 6.23 | 28.32 |
| | 2 | 2.58 | 3.19 | 6.11 | 6.18 | 27.30 |
| | 3 | 2.21 | 3.12 | 6.01 | 6.43 | 28.19 |
| K14 | 1 | 1.22 | 2.68 | 2.74 | 5.09 | 33.46 |
| | 2 | 0.95 | 2.94 | 2.40 | 5.00 | 33.09 |
| | 3 | 1.04 | 2.46 | 3.45 | 5.66 | 32.92 |
| K22 | 1 | 1.57 | 2.22 | 5.19 | 4.81 | 23.86 |
| | 2 | 1.86 | 2.23 | 6.26 | 5.89 | 27.20 |
| | 3 | 1.99 | 2.31 | 5.09 | 6.45 | 29.12 |
| L3180 | 1 | 1.14 | 3.87 | 3.16 | 5.53 | 28.80 |
| | 2 | 1.35 | 4.51 | 3.80 | 7.01 | 25.87 |
| | 3 | 0.88 | 3.97 | 3.28 | 6.23 | 33.32 |
| LIAO159 | 1 | 1.73 | 3.09 | 4.18 | 7.89 | 28.29 |
| | 2 | 1.22 | 2.66 | 6.55 | 7.44 | 27.59 |
| | 3 | 1.16 | 3.23 | 3.20 | 7.37 | 28.82 |
| LIAO5114 | 1 | 1.50 | 4.10 | 3.24 | 8.78 | 24.23 |
| | 2 | 1.01 | 4.75 | 7.33 | 9.50 | 27.94 |
| | 3 | 1.47 | 3.53 | 5.40 | 9.17 | 34.25 |
| LK11 | 1 | 0.86 | 2.56 | 3.31 | 6.45 | 34.96 |
| | 2 | 1.00 | 1.92 | 5.54 | 4.77 | 31.35 |

| | | | | | | |
|---|---|---|---|---|---|---|
| | 3 | 1.39 | 2.39 | 3.08 | 5.32 | 26.95 |
| LV28 | 1 | 0.73 | 2.92 | 6.80 | 6.41 | 28.75 |
| | 2 | 0.63 | 2.45 | 8.63 | 7.07 | 28.59 |
| | 3 | 1.16 | 3.06 | 7.21 | 5.97 | 34.10 |
| LX9801 | 1 | 1.55 | 2.03 | 4.73 | 6.04 | 30.98 |
| | 2 | 0.73 | 3.11 | 5.29 | 6.50 | 28.77 |
| | 3 | 0.87 | 2.77 | 5.11 | 5.66 | 28.19 |
| LXN | 1 | 0.88 | 3.86 | 2.57 | 6.39 | 30.46 |
| | 2 | 0.72 | 4.00 | 3.24 | 5.76 | 27.24 |
| | 3 | 0.92 | 3.62 | 4.61 | 5.64 | 26.50 |
| LY042 | 1 | 1.09 | 1.89 | 7.46 | 4.91 | 28.35 |
| | 2 | 0.75 | 1.98 | 8.24 | 4.97 | 24.65 |
| | 3 | 1.26 | 2.04 | 7.85 | 5.04 | 29.53 |
| MO113 | 1 | 0.45 | 1.82 | 3.17 | 7.38 | 23.51 |
| | 2 | 0.70 | 3.10 | 3.34 | 7.48 | 25.55 |
| | 3 | 0.75 | 2.47 | 3.73 | 7.05 | 27.54 |
| MO17 | 1 | 1.55 | 2.86 | 4.51 | 7.71 | 30.74 |
| | 2 | 1.22 | 3.68 | 5.26 | 7.06 | 32.16 |
| | 3 | 1.56 | 2.40 | 4.25 | 7.44 | 33.66 |
| NAN21-3 | 1 | 1.76 | 2.48 | 4.71 | 6.36 | 27.34 |
| | 2 | 1.12 | 3.70 | 4.67 | 7.85 | 27.14 |
| | 3 | 1.66 | 2.48 | 5.14 | 7.29 | 26.74 |
| P178 | 1 | 0.52 | 2.94 | 7.52 | 6.25 | 30.41 |
| | 2 | 0.84 | 2.74 | 6.03 | 5.57 | 28.53 |
| | 3 | 0.54 | 3.19 | 8.77 | 5.21 | 34.55 |
| QI205 | 1 | 2.44 | 2.63 | 3.81 | 4.95 | 26.10 |
| | 2 | 1.84 | 2.09 | 3.19 | 6.57 | 24.57 |
| | 3 | 2.61 | 1.54 | 3.26 | 5.92 | 25.40 |
| RY713 | 1 | 2.17 | 2.28 | 3.28 | 9.71 | 25.36 |
| | 2 | 1.80 | 3.19 | 2.54 | 8.10 | 25.35 |
| | 3 | 2.30 | 2.89 | 3.46 | 8.68 | 25.80 |
| S37 | 1 | 1.36 | 2.65 | 4.14 | 5.35 | 22.25 |
| | 2 | 1.10 | 2.59 | 4.02 | 6.61 | 21.55 |
| | 3 | 1.60 | 2.41 | 3.85 | 5.22 | 21.54 |
| SHEN5003 | 1 | 2.46 | 3.69 | 5.40 | 5.24 | 30.94 |
| | 2 | 2.65 | 3.50 | 4.98 | 6.24 | 32.42 |
| | 3 | 2.13 | 3.41 | 4.96 | 5.68 | 30.68 |
| SW92E114 | 1 | 1.10 | 2.32 | 4.72 | 5.98 | 30.36 |
| | 2 | 1.14 | 2.94 | 4.41 | 4.40 | 25.16 |
| | 3 | 1.14 | 2.70 | 3.99 | 6.97 | 27.92 |
| SY1052 | 1 | 1.14 | 2.34 | 3.62 | 7.15 | 25.94 |
| | 2 | 1.24 | 2.46 | 2.80 | 5.98 | 25.20 |
| | 3 | 0.91 | 2.48 | 3.03 | 8.16 | 26.43 |
| SY3073 | 1 | 0.74 | 3.56 | 2.80 | 6.21 | 25.46 |

| | | | | | | |
|---|---|---|---|---|---|---|
| | 2 | 0.56 | 4.88 | 2.71 | 6.53 | 25.92 |
| | 3 | 0.76 | 4.52 | 3.26 | 5.96 | 25.22 |
| TIAN77 | 1 | 2.22 | 2.75 | 3.27 | 6.42 | 25.73 |
| | 2 | 1.94 | 2.80 | 3.50 | 6.75 | 24.96 |
| | 3 | 1.42 | 2.93 | 3.31 | 6.50 | 25.61 |
| TIE7922 | 1 | 1.47 | 3.50 | 4.05 | 4.59 | 24.80 |
| | 2 | 1.04 | 3.44 | 5.47 | 5.41 | 28.04 |
| | 3 | 1.30 | 3.02 | 3.45 | 6.04 | 29.19 |
| TY1 | 1 | 0.86 | 3.22 | 3.95 | 7.04 | 29.12 |
| | 2 | 1.53 | 2.99 | 4.75 | 6.78 | 31.23 |
| | 3 | 1.37 | 2.82 | 4.02 | 8.44 | 31.18 |
| TY11 | 1 | 0.91 | 3.56 | 3.76 | 5.48 | 30.14 |
| | 2 | 0.57 | 2.85 | 5.77 | 5.82 | 28.40 |
| | 3 | 0.79 | 2.96 | 3.37 | 6.10 | 27.50 |
| TY2 | 1 | 0.76 | 3.02 | 3.93 | 5.96 | 31.83 |
| | 2 | 0.57 | 3.73 | 2.39 | 5.78 | 24.17 |
| | 3 | 0.76 | 2.92 | 4.22 | 6.14 | 25.93 |
| TY3 | 1 | 0.92 | 3.88 | 5.45 | 4.18 | 20.03 |
| | 2 | 1.42 | 4.43 | 4.48 | 5.16 | 20.00 |
| | 3 | 1.59 | 3.96 | 5.08 | 5.34 | 20.29 |
| TY4 | 1 | 0.86 | 2.71 | 3.17 | 5.07 | 34.71 |
| | 2 | 0.64 | 2.86 | 7.20 | 5.91 | 28.38 |
| | 3 | 0.79 | 3.32 | 4.33 | 6.48 | 27.01 |
| TY5 | 1 | 1.10 | 2.26 | 4.00 | 5.77 | 28.79 |
| | 2 | 0.74 | 2.88 | 2.83 | 5.50 | 27.55 |
| | 3 | 0.83 | 2.59 | 3.50 | 5.82 | 29.92 |
| TY6 | 1 | 0.86 | 2.65 | 2.90 | 7.12 | 27.09 |
| | 2 | 0.98 | 2.34 | 2.87 | 7.37 | 24.35 |
| | 3 | 0.75 | 2.91 | 2.92 | 6.99 | 33.50 |
| U8112 | 1 | 0.65 | 3.70 | 3.81 | 6.16 | 26.20 |
| | 2 | 0.62 | 4.18 | 6.70 | 6.12 | 26.01 |
| | 3 | 0.54 | 4.35 | 4.12 | 6.90 | 28.40 |
| W138 | 1 | 1.00 | 3.24 | 6.43 | 7.88 | 26.76 |
| | 2 | 1.04 | 3.38 | 4.61 | 7.31 | 32.03 |
| | 3 | 1.10 | 2.58 | 4.65 | 7.79 | 31.95 |
| WH413 | 1 | 1.54 | 2.82 | 3.23 | 5.59 | 26.30 |
| | 2 | 1.26 | 3.15 | 3.07 | 6.10 | 27.09 |
| | 3 | 1.31 | 3.25 | 3.85 | 7.15 | 25.76 |
| WU109 | 1 | 1.23 | 2.72 | 4.03 | 6.05 | 26.24 |
| | 2 | 1.08 | 2.86 | 3.65 | 7.46 | 22.87 |
| | 3 | 1.13 | 2.46 | 4.41 | 6.34 | 24.93 |
| XI502 | 1 | 0.79 | 2.63 | 3.69 | 6.36 | 22.48 |
| | 2 | 0.78 | 2.29 | 2.79 | 5.74 | 22.50 |
| | 3 | 0.92 | 2.38 | 3.65 | 7.06 | 22.72 |

| | | | | | | |
|---|---|---|---|---|---|---|
| XUN971 | 1 | 0.39 | 3.53 | 4.87 | 5.04 | 30.65 |
| | 2 | 0.22 | 3.11 | 3.98 | 5.66 | 34.63 |
| | 3 | 0.31 | 3.06 | 5.61 | 6.49 | 30.76 |
| YE478 | 1 | 1.49 | 3.74 | 3.92 | 7.00 | 28.97 |
| | 2 | 0.85 | 3.12 | 2.70 | 7.11 | 28.09 |
| | 3 | 1.43 | 3.15 | 3.23 | 8.00 | 26.78 |
| YE52106 | 1 | 1.15 | 2.23 | 3.80 | 7.11 | 31.67 |
| | 2 | 1.79 | 2.87 | 4.55 | 8.13 | 27.81 |
| | 3 | 0.93 | 2.74 | 4.58 | 6.97 | 26.78 |
| YE8001 | 1 | 2.88 | 3.14 | 4.51 | 7.91 | 32.24 |
| | 2 | 2.42 | 2.48 | 3.87 | 7.97 | 22.25 |
| | 3 | 1.75 | 2.63 | 4.98 | 8.58 | 29.90 |
| Z2018F | 1 | 2.25 | 1.95 | 4.17 | 8.89 | 26.94 |
| | 2 | 2.43 | 2.06 | 5.32 | 8.63 | 26.59 |
| | 3 | 2.28 | 2.33 | 4.03 | 8.44 | 28.51 |
| ZHENG28 | 1 | 1.91 | 2.65 | 3.22 | 5.19 | 31.93 |
| | 2 | 2.75 | 2.82 | 5.84 | 6.54 | 29.99 |
| | 3 | 1.36 | 2.53 | 3.66 | 6.63 | 31.67 |
| ZHENG29 | 1 | 0.73 | 2.33 | 2.53 | 6.06 | 30.89 |
| | 2 | 0.77 | 2.79 | 1.55 | 6.14 | 26.28 |
| | 3 | 1.45 | 3.94 | 2.82 | 7.23 | 31.91 |
| ZHENG35 | 1 | 0.96 | 2.46 | 6.57 | 5.65 | 28.30 |
| | 2 | 1.07 | 1.81 | 6.31 | 5.62 | 25.91 |
| | 3 | 0.87 | 2.07 | 6.34 | 6.30 | 27.88 |
| ZHENG653 | 1 | 0.62 | 2.90 | 4.72 | 7.03 | 32.00 |
| | 2 | 0.69 | 2.98 | 4.42 | 6.78 | 31.29 |
| | 3 | 0.78 | 2.94 | 4.68 | 6.58 | 31.99 |
| ZHONG69 | 1 | 1.05 | 2.84 | 3.56 | 5.58 | 25.30 |
| | 2 | 0.94 | 3.42 | 2.73 | 5.80 | 24.94 |
| | 3 | 1.07 | 3.43 | 4.08 | 5.88 | 25.10 |
| ZONG31 | 1 | 1.16 | 3.21 | 2.39 | 7.34 | 28.67 |
| | 2 | 0.57 | 3.30 | 3.97 | 6.62 | 32.73 |
| | 3 | 0.98 | 3.39 | 4.76 | 7.36 | 28.55 |
| ZZ01 | 1 | 2.21 | 1.93 | 3.53 | 5.75 | 32.44 |
| | 2 | 2.25 | 1.80 | 3.69 | 6.62 | 27.84 |
| | 3 | 2.10 | 2.66 | 4.24 | 5.48 | 25.73 |
| ZZ03 | 1 | 1.28 | 2.15 | 4.84 | 5.29 | 24.81 |
| | 2 | 1.82 | 2.86 | 4.36 | 5.91 | 25.22 |
| | 3 | 2.29 | 3.12 | 5.58 | 4.62 | 27.14 |
| 501 | 1 | 0.88 | 3.19 | 5.48 | 6.23 | 32.07 |
| | 2 | 0.54 | 3.58 | 5.65 | 6.33 | 29.98 |
| | 3 | 0.94 | 3.22 | 5.43 | 6.54 | 32.89 |
| 812 | 1 | 0.96 | 2.36 | 4.76 | 7.08 | 27.08 |
| | 2 | 0.77 | 4.08 | 4.92 | 6.33 | 24.59 |

| | | | | | | |
|---|---|---|---|---|---|---|
| | 3 | 1.05 | 3.49 | 5.78 | 6.96 | 25.92 |
| 1323 | 1 | 0.63 | 2.53 | 2.21 | 4.20 | 30.18 |
| | 2 | 0.86 | 2.27 | 2.42 | 4.85 | 28.46 |
| | 3 | 0.95 | 2.18 | 3.09 | 5.10 | 27.84 |
| 3411 | 1 | 0.79 | 3.88 | 5.53 | 5.77 | 27.05 |
| | 2 | 1.47 | 3.46 | 5.45 | 6.98 | 25.76 |
| | 3 | 0.69 | 3.06 | 5.78 | 6.02 | 28.39 |
| 04K5672 | 1 | 0.46 | 2.54 | 3.79 | 5.24 | 35.62 |
| | 2 | 0.55 | 2.90 | 4.58 | 5.54 | 30.44 |
| | 3 | 0.75 | 3.78 | 3.25 | 5.19 | 29.27 |
| 04K5702 | 1 | 0.86 | 2.41 | 5.20 | 6.01 | 27.14 |
| | 2 | 1.01 | 2.67 | 5.87 | 5.91 | 26.64 |
| | 3 | 1.06 | 2.78 | 4.16 | 6.89 | 30.76 |
| 384-2 | 1 | 1.95 | 2.65 | 4.32 | 5.41 | 28.06 |
| | 2 | 1.19 | 2.72 | 4.81 | 5.83 | 27.87 |
| | 3 | 1.38 | 2.48 | 4.43 | 4.39 | 26.96 |
| BGY | 1 | 1.53 | 2.95 | 3.46 | 6.68 | 27.02 |
| | 2 | 1.15 | 4.38 | 4.49 | 6.84 | 27.19 |
| | 3 | 1.13 | 3.34 | 3.79 | 6.20 | 28.56 |
| BY843 | 1 | 1.65 | 5.24 | 5.13 | 5.31 | 28.19 |
| | 2 | 2.01 | 2.29 | 4.18 | 6.13 | 29.27 |
| | 3 | 2.26 | 1.94 | 4.99 | 7.28 | 34.90 |
| CF3 | 1 | 0.75 | 3.47 | 3.84 | 7.34 | 31.59 |
| | 2 | 1.07 | 2.25 | 3.73 | 6.43 | 29.49 |
| | 3 | 0.99 | 2.80 | 2.61 | 6.19 | 30.96 |
| CIMBL110 | 1 | 2.92 | 1.99 | 4.60 | 4.58 | 28.61 |
| | 2 | 2.48 | 1.90 | 4.01 | 5.04 | 29.28 |
| | 3 | 2.36 | 2.14 | 4.60 | 5.05 | 27.66 |
| CIMBL112 | 1 | 1.06 | 2.11 | 4.10 | 9.51 | 27.45 |
| | 2 | 1.33 | 2.48 | 3.83 | 5.16 | 24.97 |
| | 3 | 1.22 | 2.58 | 4.25 | 5.00 | 24.70 |
| CIMBL117 | 1 | 0.53 | 2.13 | 2.99 | 4.53 | 30.49 |
| | 2 | 0.47 | 2.23 | 5.29 | 3.73 | 29.05 |
| | 3 | 0.90 | 2.91 | 3.19 | 4.43 | 32.63 |
| CIMBL134 | 1 | 1.01 | 1.85 | 4.14 | 5.51 | 25.46 |
| | 2 | 0.47 | 3.59 | 4.34 | 5.69 | 24.68 |
| | 3 | 0.59 | 2.97 | 5.03 | 7.03 | 29.21 |
| CIMBL136 | 1 | 1.60 | 2.74 | 2.74 | 6.76 | 27.71 |
| | 2 | 0.97 | 2.47 | 2.17 | 4.52 | 26.54 |
| | 3 | 1.84 | 2.51 | 2.36 | 5.81 | 27.92 |
| CIMBL138 | 1 | 1.48 | 3.27 | 2.60 | 6.88 | 26.64 |
| | 2 | 2.17 | 3.33 | 3.23 | 5.56 | 25.02 |
| | 3 | 1.55 | 2.46 | 3.44 | 6.88 | 22.19 |
| CIMBL146 | 1 | 1.93 | 2.88 | 4.33 | 6.30 | 29.08 |

| | | | | | | |
|---|---|---|---|---|---|---|
| | 2 | 1.61 | 3.36 | 4.95 | 6.27 | 26.92 |
| | 3 | 1.44 | 3.59 | 5.17 | 6.98 | 26.49 |
| CIMBL154 | 1 | 1.24 | 2.65 | 5.90 | 6.43 | 29.67 |
| | 2 | 1.64 | 2.94 | 7.63 | 5.96 | 31.35 |
| | 3 | 1.52 | 2.79 | 6.30 | 6.57 | 31.22 |
| CIMBL30 | 1 | 1.39 | 3.97 | 4.44 | 5.56 | 29.60 |
| | 2 | 0.95 | 2.47 | 9.09 | 5.33 | 28.35 |
| | 3 | 0.68 | 2.54 | 4.20 | 4.36 | 31.93 |
| CIMBL34 | 1 | 2.69 | 2.34 | 4.80 | 7.06 | 28.72 |
| | 2 | 2.67 | 3.30 | 4.90 | 6.22 | 26.99 |
| | 3 | 2.16 | 3.32 | 4.89 | 6.14 | 28.01 |
| CIMBL45 | 1 | 1.41 | 2.65 | 6.07 | 5.88 | 37.32 |
| | 2 | 0.36 | 3.19 | 8.06 | 6.78 | 36.13 |
| | 3 | 0.36 | 2.17 | 6.29 | 6.03 | 36.62 |
| CIMBL57 | 1 | 1.47 | 2.21 | 5.81 | 4.77 | 24.41 |
| | 2 | 1.30 | 3.25 | 5.80 | 5.96 | 22.42 |
| | 3 | 1.50 | 3.58 | 5.72 | 6.51 | 26.66 |
| CIMBL67 | 1 | 1.88 | 2.73 | 5.11 | 6.30 | 29.67 |
| | 2 | 1.43 | 1.63 | 5.85 | 5.09 | 28.78 |
| | 3 | 1.40 | 2.53 | 5.35 | 4.75 | 27.93 |
| CIMBL8 | 1 | 0.66 | 3.56 | 3.04 | 8.27 | 26.85 |
| | 2 | 0.43 | 4.39 | 4.10 | 6.83 | 27.51 |
| | 3 | 0.22 | 4.65 | 2.98 | 5.73 | 26.21 |
| CIMBL80 | 1 | 1.00 | 1.91 | 3.03 | 5.55 | 26.99 |
| | 2 | 0.71 | 2.89 | 5.76 | 5.06 | 25.08 |
| | 3 | 1.35 | 2.83 | 5.19 | 6.15 | 24.25 |
| CIMBL85 | 1 | 0.91 | 3.56 | 4.31 | 5.54 | 35.68 |
| | 2 | 1.47 | 2.69 | 5.43 | 6.64 | 32.14 |
| | 3 | 0.75 | 3.75 | 5.35 | 6.17 | 35.44 |
| D047 | 1 | 1.42 | 3.36 | 3.71 | 5.78 | 33.03 |
| | 2 | 1.66 | 2.90 | 4.13 | 5.27 | 32.46 |
| | 3 | 1.09 | 3.12 | 2.89 | 6.03 | 32.28 |
| DAN598 | 1 | 1.49 | 2.98 | 2.82 | 5.83 | 29.21 |
| | 2 | 0.92 | 3.61 | 3.24 | 5.86 | 26.89 |
| | 3 | 1.05 | 3.44 | 2.73 | 6.08 | 28.76 |
| DH29 | 1 | 0.91 | 2.17 | 4.30 | 6.98 | 21.68 |
| | 2 | 1.13 | 2.35 | 5.23 | 6.38 | 25.20 |
| | 3 | 1.15 | 2.38 | 4.49 | 7.33 | 24.62 |
| DSB | 1 | 1.38 | 4.26 | 3.82 | 4.50 | 25.32 |
| | 2 | 0.86 | 3.63 | 4.87 | 4.81 | 27.13 |
| | 3 | 1.28 | 4.05 | 3.63 | 5.17 | 25.43 |
| GEMS12 | 1 | 1.36 | 2.38 | 3.89 | 7.01 | 29.61 |
| | 2 | 1.71 | 2.53 | 3.83 | 7.94 | 25.65 |
| | 3 | 2.44 | 2.25 | 4.40 | 8.84 | 25.56 |

| | | | | | | |
|---|---|---|---|---|---|---|
| GEMS27 | 1 | 0.72 | 2.71 | 4.28 | 5.03 | 27.83 |
| | 2 | 1.13 | 3.25 | 6.61 | 3.94 | 27.73 |
| | 3 | 0.91 | 3.25 | 3.80 | 5.13 | 30.90 |
| GEMS53 | 1 | 1.30 | 1.87 | 6.64 | 6.18 | 26.97 |
| | 2 | 1.54 | 3.42 | 6.26 | 6.55 | 27.55 |
| | 3 | 1.82 | 2.57 | 6.75 | 6.05 | 25.90 |
| GY220 | 1 | 0.83 | 2.71 | 2.67 | 12.30 | 35.07 |
| | 2 | 1.11 | 2.88 | 4.25 | 9.68 | 37.91 |
| | 3 | 1.30 | 2.61 | 3.94 | 6.97 | 37.13 |
| GY237 | 1 | 0.90 | 2.34 | 4.39 | 5.99 | 29.26 |
| | 2 | 0.64 | 3.52 | 4.84 | 5.72 | 20.85 |
| | 3 | 0.80 | 3.55 | 4.53 | 5.17 | 24.71 |
| GY246 | 1 | 1.63 | 2.27 | 4.98 | 7.17 | 27.99 |
| | 2 | 1.26 | 3.37 | 3.17 | 6.85 | 25.14 |
| | 3 | 1.18 | 2.74 | 4.02 | 7.35 | 26.31 |
| HB | 1 | 0.98 | 2.68 | 4.39 | 8.05 | 31.35 |
| | 2 | 1.47 | 3.15 | 3.29 | 5.73 | 24.69 |
| | 3 | 0.43 | 3.26 | 3.79 | 6.63 | 28.39 |
| HU803 | 1 | 1.81 | 3.27 | 3.93 | 6.69 | 24.78 |
| | 2 | 1.54 | 3.89 | 3.65 | 6.40 | 22.54 |
| | 3 | 1.37 | 3.57 | 3.74 | 7.11 | 26.22 |
| M165 | 1 | 0.62 | 2.49 | 5.68 | 8.78 | 29.62 |
| | 2 | 0.56 | 2.63 | 8.59 | 7.61 | 32.25 |
| | 3 | 0.66 | 2.34 | 6.77 | 8.42 | 31.35 |
| MN | 1 | 1.49 | 3.70 | 3.48 | 5.78 | 28.27 |
| | 2 | 1.44 | 3.69 | 4.43 | 6.78 | 26.71 |
| | 3 | 1.76 | 3.69 | 4.03 | 6.50 | 26.43 |
| QI319 | 1 | 1.20 | 2.05 | 4.88 | 8.74 | 26.53 |
| | 2 | 1.18 | 2.40 | 5.14 | 9.06 | 32.69 |
| | 3 | 1.44 | 2.87 | 5.55 | 9.13 | 29.31 |
| R08 | 1 | 2.27 | 3.64 | 4.78 | 4.68 | 25.85 |
| | 2 | 1.18 | 3.18 | 4.80 | 6.63 | 23.84 |
| | 3 | 1.25 | 3.47 | 4.23 | 6.04 | 27.82 |
| SHEN137 | 1 | 1.64 | 2.89 | 3.08 | 7.48 | 30.09 |
| | 2 | 0.92 | 2.85 | 2.18 | 7.29 | 29.84 |
| | 3 | 1.26 | 2.69 | 3.31 | 7.09 | 31.32 |
| SI444 | 1 | 1.64 | 3.06 | 3.69 | 6.63 | 32.30 |
| | 2 | 0.85 | 2.75 | 3.52 | 8.33 | 30.85 |
| | 3 | 1.13 | 3.58 | 3.41 | 7.74 | 31.16 |
| SY1077 | 1 | 1.05 | 3.14 | 5.77 | 10.74 | 26.36 |
| | 2 | 1.07 | 3.16 | 5.34 | 10.27 | 25.50 |
| | 3 | 0.72 | 3.15 | 5.06 | 10.46 | 25.54 |
| SY999 | 1 | 2.47 | 2.47 | 4.29 | 8.27 | 26.66 |
| | 2 | 3.11 | 2.65 | 6.27 | 8.70 | 24.78 |

| | | | | | | |
|---|---|---|---|---|---|---|
| | 3 | 1.59 | 2.58 | 4.29 | 8.28 | 28.29 |
| TT16 | 1 | 1.01 | 2.22 | 2.87 | 5.94 | 27.69 |
| | 2 | 1.10 | 3.49 | 4.11 | 6.46 | 28.93 |
| | 3 | 1.15 | 2.49 | 3.22 | 6.34 | 24.87 |
| TY10 | 1 | 1.75 | 1.83 | 4.37 | 6.11 | 30.17 |
| | 2 | 1.83 | 2.79 | 3.66 | 6.38 | 29.04 |
| | 3 | 1.53 | 2.72 | 4.37 | 5.00 | 30.46 |
| TY7 | 1 | 1.57 | 2.99 | 4.21 | 5.58 | 23.74 |
| | 2 | 1.41 | 2.88 | 4.98 | 6.47 | 26.64 |
| | 3 | 1.84 | 2.96 | 4.42 | 5.87 | 22.54 |
| TY8 | 1 | 1.14 | 1.24 | 3.51 | 6.35 | 28.83 |
| | 2 | 1.78 | 2.01 | 3.21 | 7.47 | 32.82 |
| | 3 | 1.51 | 2.62 | 3.62 | 8.17 | 30.07 |
| WMR | 1 | 2.23 | 2.92 | 4.05 | 6.41 | 30.06 |
| | 2 | 2.39 | 3.73 | 5.10 | 5.73 | 24.94 |
| | 3 | 2.43 | 3.28 | 4.55 | 6.03 | 28.10 |
| ZHENG58 | 1 | 0.46 | 2.42 | 5.06 | 5.81 | 29.34 |
| | 2 | 0.45 | 3.37 | 5.27 | 5.15 | 32.68 |
| | 3 | 0.48 | 3.06 | 5.28 | 6.44 | 32.90 |

1,2,3 three biological repeats.
